# Synthesis of new sulfoximines and sulfonimidamides derivatives as NKCC1 inhibitors

**DOI:** 10.3389/fchem.2026.1804888

**Published:** 2026-07-02

**Authors:** Gerald Coste, Vincent Rodeschini, Pascal George, Eric Delpire, Yehezkel Ben-Ari

**Affiliations:** 1 Edelris, Lyon, France; 2 Drug Discovery Kagakuteki Consulting D2KC SAS, Longvilliers, France; 3 Department of Anesthesiology, Vanderbilt University School of Medicine, Nashville, TN, United States; 4 Ba-Oncomedical, Marseille, France; 5 Neurochlore, Marseille, France

**Keywords:** bumetanide, K–Cl cotransporter, Na–K–2Cl cotransporter, sulfonamides, sulfonimidamide, sulfoximine

## Abstract

**Introduction:**

The Na–K–2Cl cotransporter isoform 1 (NKCC1) regulates cell volume and ionic distribution, thereby also controlling the efficacy of neuronal GABAergic inhibition. enhanced activity of the NKCC1 chloride importer and high Cl. levels has been reported in a long list of disorders, including epilepsies, brain trauma, peripheral and central cancers, spinal cord injury or chronic pain, and cerebrovascular infarcts, indicating that NKCC1 inhibitors might constitute promising therapeutic avenues. Synthetized four decades ago, bumetanide constitutes the only agent that is widely used in animal models and human trials, limiting the possibility to assign some NKCC1 inhibitors specifically to treat some disorders.

**Methods:**

Here, we have synthetized many novel NKCC1 inhibitors to augment the range of molecules that can be tested in animal models and pre-IND tests. We used modifications of the bumetanide parent molecule by modifying two sites with alterations that have not been envisaged before. Specifically, we incorporated carboxylic acid bioisosteres and sulfonimidamides or sulfoximines moieties. Their efficacy was evaluated against NKCC1, NKCC2, and KCC2 using human cell lines.

**Results and discussion:**

The synthesis of the new inhibitors and the structure–activity relationship (SAR) are described. Some molecules are superior to bumetanide as NKCC1 inhibitors, which widens the family of NKCC1 inhibitors and paves the way for more efficient agents. Our aims here are solely chemical, namely, describing the possible targets of the bumetanide molecule that can be changed using novel approaches. In the future, we and others will test these molecules in animal models and clinical trials to validate the use of some of them in treating a variety of disorders.

## 1 Introduction

The Na–K–2Cl cotransporter (NKCC) is a transport protein that mediates the secondary active transport of sodium, potassium, and chloride into cells. Two isoforms of this membrane transport protein are present in humans, namely, NKCC1 and NKCC2. In neurons in physiological conditions, NKCC1 imports ions, particularly chloride, which plays a crucial role in maintaining the chloride levels. This is balanced by KCC2, which exports ions, notably chloride (Delpire, 2000; Rivera et al., 1999). The equilibrium between these two cotransporters is paramount in controlling neuronal excitability and cell volume. This also controls GABAergic inhibition, which is highly dependent on neuronal chloride levels. Low levels of neuronal chloride are associated with inhibition, while high levels are usually associated with depolarization and excitation, along with changes in cell volume. Extensive analyses indicate that pathological conditions are associated with the disruption of the regulation of ionic distributions in neurons and non-neuronal cells due to a disequilibrium between the activity of the cotransporters NKCC1 and KCC2 (Ben-Ari, 2017; Garzon-Muvdi et al., 2012; Habela et al., 2009; Hasbargen et al., 2010; Nabekura et al., 2002; Savardi et al., 2020).

In many pathological conditions, NKCC1 activity is upregulated and KCC2 activity is downregulated, leading to excitatory actions of GABA and epileptic activity (Ben-Ari, 2017; Cohen et al., 2002; MacKenzie et al., 2016; Savardi et al., 2021). This has been observed in epilepsies, autism, Rett syndrome, and fragile X syndrome, and it also observed in ischemic and traumatic insults, chronic pain, and a large variety of cancers, including glioblastomas, (Ben-Ari, 2017; Dzhala et al., 2010; Garzon-Muvdi et al., 2012; Habela et al., 2009; Hasbargen et al., 2010; Hyde et al., 2011; Lozovaya et al., 2018; Nardou et al., 2011; Savardi et al., 2021). To the best of our knowledge, there are very few molecules that have such a large amount of promising animal and human data. This has led to renewed interest in the research and development of NKCC1 antagonists (and/or KCC2 enhancers) to treat disorders (Savardi et al., 2020).

Bumetanide, an FDA-approved NKCC1 inhibitor used as a diuretic agent, was first discovered in 1969 at Leo Pharma through phenotypic screening (Feit, 1971; Feit, 1981). It was later demonstrated that the bumetanide diuretic effect is correlated with NKCC inhibition (Eveloff and Kinne, 1983; Forbush and Palfrey, 1983). Bumetanide accelerates temozolomide-mediated apoptosis (Algharabil et al., 2012) and reduces cell migration glioma cell invasion (Ma et al., 2019; Zhu et al., 2014), and NKCC1 activity promotes the activity of temozolomide (Luo et al., 2020). Bumetanide and NKCC1 inhibition have been shown to be efficient in colon cancers (Malamas et al., 2015), hepato-cellular carcinoma (Yarmohammadi et al., 2017; Zhou et al., 2017), small cell lung cancers (Sun et al., 2016), prostate cancers (Hiraoka et al., 2010), and gastric cancer cells (Wang et al., 2021). NKCC1 activity predicts poor prognosis in lung adenocarcinoma (Sun et al., 2016). More recently, bumetanide has been evaluated in animal models and clinical trials for epilepsies and autism, reaching phase 3 for the latter (Delpire and Ben-Ari, 2022; Fuentes et al., 2023; Lemonnier et al., 2017; Rabiei et al., 2026; Xiao et al., 2024). Similarly, bumetanide was shown to be effective in blocking seizures in both human temporal lobe epilepsies in two phase-2 trials (Gharaylou et al., 2019; Soul et al., 2021) and in human surgically removed temporal lobe epilepsies (Huberfeld et al., 2008). Bumetanide also attenuates autism syndromes but not seizures in adolescents with tuberous sclerosis (van Andel et al., 2020). Bumetanide was found to be the best among 1,000 widely used agents in blocking APOE-dependent Alzheimer’s disease preparations (Taubes et al., 2021). In addition, in an epidemiological study on 4 million US citizen aged over 65 years, the use of bumetanide reduced the likelihood of Alzheimer’s disease by 30%–70%. Recent studies also indicate that the failure to treat brain tumors is largely due to the extensive reactive plasticity taking place, leading to the establishment of aberrant synaptic connections between the tumors and their neighborhood. This, in turn, leads to enhanced hyperactivity and seizures due to the enhanced NKCC1 activity and excitatory actions of GABA and hyperactivity of the excitatory glutamatergic synapses (Barron et al., 2025; Beichert et al., 2025; Elias et al., 2023; Hanahan and Monje, 2023; Huang-Hobbs et al., 2023; Jung et al., 2019; Venkataramani et al., 2019; Venkataramani et al., 2022). This has been validated in surgically removed isolated glioblastoma tumoral tissue (Barron et al., 2025; Bourgeois et al., 2025).

**FIGURE 1:**
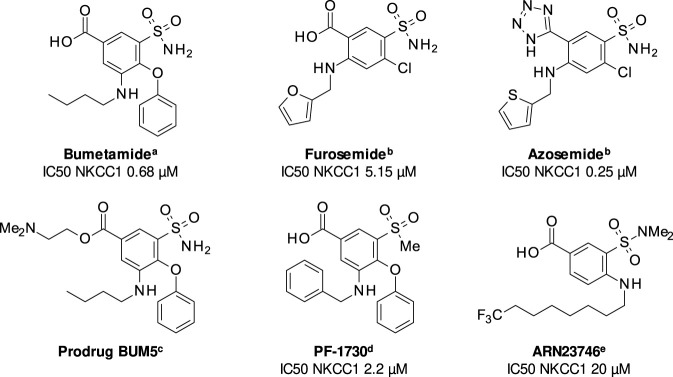
Representative list of NKCC1 inhibitors reported in the scientific literature. **(a)** Lykke et al. (2016); **(b)** Hampel et al. (2018); **(c)** Töllner et al. (2014); **(d)** Lykke et al. (2015); **(e)** Savardi et al. (2020).

In view of such a wide range of peripheral and central disorders in which NKCC1 inhibition is efficient, it appeared mandatory to develop novel analogs to widen the range of disorders that can be treated with NKCC1 inhibitors and compensate the likely failure of some of the analogs in future pre-IND tests aimed at testing these agents in patients. Specifically, our aim here is not to suggest a treatment for a particular disorder but to offer a wide range of proprietary NKCC1 inhibitors to stimulate research on its role in brain and peripheral disorders. These molecules will be available for experimental studies by teams interested in pursuing their own research, to enrich our knowledge on their actions, in parallel to our own clinical trials centered on brain tumors, autism, and epilepsies. Several studies have aimed to identify new compounds with enhanced activity or improved physicochemical properties than bumetanide using approaches such as SAR analysis (Lykke et al., 2015), high-throughput screening (HTS) (Gill et al., 2017), virtual screening (Roy et al., 2021), or prodrug strategies (Töllner et al., 2014) (Figure 1). Recently, Savardi et al. (2020) developed a pharmacophore model using bumetanide and other NKCC1 inhibitors through a ligand-based computational method and reported the identification of a new compound, ARN23746, with an IC_50_ of 20 µM against NKCC1, limited brain penetration, and no diuretic side effect.

In the present work, we report the design, synthesis, and evaluation of novel NKCC1 inhibitors, aiming to identify novel drug candidates with controlled physicochemical properties that might be suitable for therapeutic applications. This paper is centered only on the chemical aspects, leaving aside for the moment the actions of these molecules on biological or therapeutic actions.

## 2 Results

### 2.1 Chemical synthesis

#### 2.1.1 Bumetanide modifications

**FIGURE 2:**
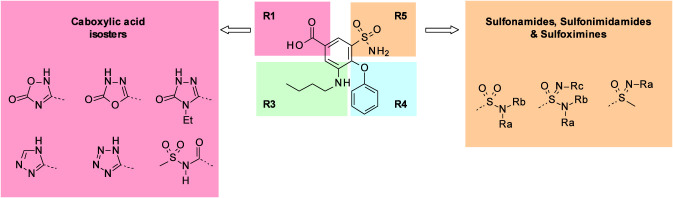
Structural modifications and numbering of bumetanide according to Hannaert et al. (2002).

The modification of the bumetanide scaffold at positions R1 and R5 was the focus of our efforts (Figure 2). Carboxylic acid alteration and prodrug strategies (R1) have been reported previously with limited success (Auer et al., 2020; Huang et al., 2019). The identification of NKCC1 inhibitors lacking a carboxylic acid group remains of high importance as this functional group has a strong impact on the physicochemical and ADMET parameters due to its ionization under physiological pH. Isosteric replacement of carboxylic acid with heteroaromatic rings is a point of entry to a largely underexplored chemical space on bumetanide analogs (Ibrahim et al., 2020; Wanaksi et al., 2010). We prepared compounds with oxadiazolone, triazolone, triazole, and tetrazole as replacements for the carboxylic acid moiety (Figure 2). To further expand this series, an *N*-acylsulfonamide, which is commonly used in medicinal chemistry as a carboxylic acid bioisoster, was also prepared (Francisco et al., 2021).

Concerning the modifications of R5, although we previously reported substituted sulfones and sulfonamides (Bourgeois et al., 2025), the introduction of other sulfur-containing groups, such as sulfoximine and sulfonimidamide, remains unexplored. Previously overlooked in medicinal chemistry, these functional groups are now gaining attention due to their versatile properties (Chinthakindi et al., 2017; Chinthakindi et al., 2019; Han et al., 2021; Luisi and Bull, 2023; Mäder and Kattner, 2020; Nandi and Arvidsson, 2018; Sehgelmeble et al., 2012; Tilby et al., 2021). They are being incorporated into various bioactive compounds in drug-discovery programs and clinical trials, particularly because of their ability to modulate the physicochemical properties and metabolic stability. Both the functional groups introduce a three-dimensional framework centered on a stereogenic sulfur atom, providing additional vectors for substitution. In our case, they are expected to lead to significant changes in the newly prepared probes, as they will alter the number of hydrogen donors and acceptors surrounding the scaffold, thus potentially improving the overall physicochemical properties and target affinity. A wide range of substituted sulfoximines and sulfonimidamides were prepared (Figure 2).

#### 2.1.2 Preparation of carboxylic acid bioisosters

**SCHEME 1:**
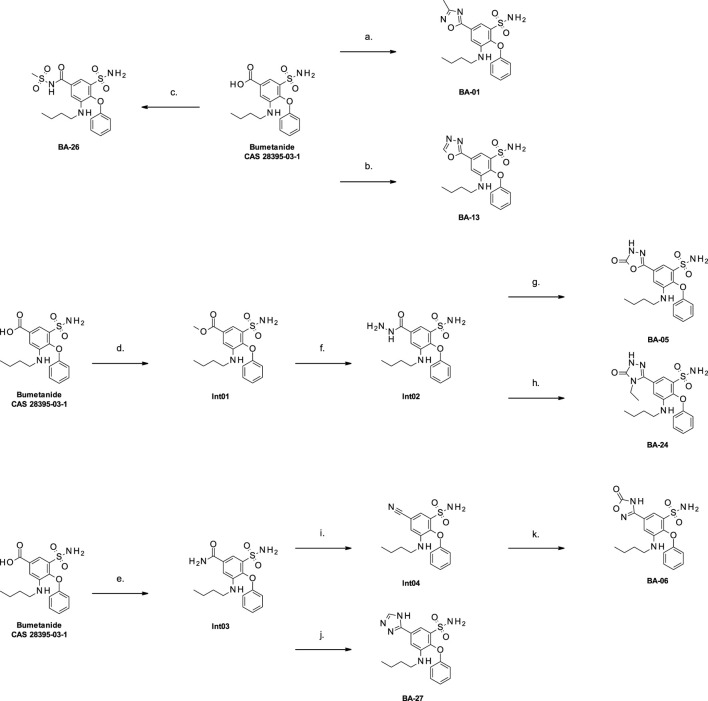
Preparation of carboxylic acid isosters. Reagents and conditions: **(a)** i. CDI, CH_3_CN, 20 °C; ii. *N*-hydroxyacetamidine; iii. Pyridine, 110 °C; **(b)** (isocyanoimino)triphenylphosphorane, DCE, 20 °C; **(c)** i. CDI, CH_3_CN, 50 °C; ii. MeSO2NH2, DBU, 20 °C; **(d)** H_2_SO_4_, MeOH, 65 °C; **(e)** i. CDI, CH_3_CN, 50 °C; ii. NH_4_OH, 20 °C; **(f)** H_2_N–NH_2_ 1M in THF, THF, 70 °C; **(g)** CDI, Et_3_N, DMF, 20 °C; **(h)** i. EtNCO, THF, 20 °C; ii. NaOH aq. 1M, 90 °C; **(i)** TFAA, pyridine, dioxane, 20 °C; **(j)** i. DMF-DMA, 20 °C; ii. H_2_N–NH_2_, AcOH, 90 °C; iii. NaOH, THF, MeOH, 20 °C; **(k)** i. NH_2_OH.HCl, NaHCO_3_, EtOH, 80 °C; ii. CDI, DBU, dioxane, 100 °C.

**SCHEME 2:**
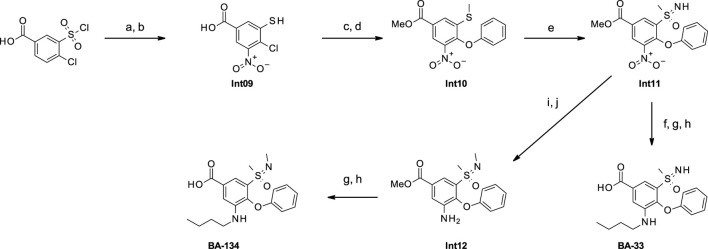
Synthesis of sulfoximines BA-134 and BA-33. Reagents and conditions: **(a)** KNO_3_, H_2_SO_4_, 100 °C; **(b)** PPh_3_, PhMe, 20 °C; **(c)** MeI, K_2_CO_3_, DMF, 20 °C; **(d)** PhOH, K_2_CO_3_, DMF, 100 °C; **(e)** PIDA, ammonium carbamate, MeOH, 20 °C; **(f)** Pd/C, H_2_ 1 atm., MeOH, 20 °C; **(g)** n-PrCHO, NaBH(OAc)_3_, DCM, 20 °C; **(h)** NaOH, THF, 20 °C; **(i)** HCOOH, HCHO, 100 °C; **(j)** NH_4_Cl, Fe, H_2_O/EtOH 1/2, 85 °C.

As described in Scheme 1, 1,2,4-oxadiazole (BA-01) was prepared by classical condensation of amidoximes with bumetanide. 1,3,4-Oxadiazole (BA-13) was conveniently prepared using (isocyanoimino)triphenylphosphorane directly on bumetanide (Nasrabadi et al., 2011).

1,3,4-Oxadiazol-2-one (BA-05) was obtained after the activation of carbazide Int02 with CDI followed by intramolecular elimination (Wurtz et al., 2022). BA-24 was obtained by reacting ethyl isocyanate with carbazide Int02, followed by an intramolecular cyclization–elimination reaction under basic conditions (Mullican et al., 1993). A streamlined synthetic pathway was designed and fine-tuned to produce compounds BA-06 and BA-27 utilizing the intermediate precursor Int03. Bumetanide was reacted with CDI and ammonium hydroxide to yield a primary amide Int03. To obtain 1,2,4-oxadiazol-5-one isomer (BA-06), the amide was dehydrated with TFAA into the corresponding nitrile Int04. Intermolecular addition of hydroxylamine on nitrile and the subsequent reaction with CDI generated BA-06 (Weaver et al., 2020). The 1,2,4-triazole (BA-27) was prepared from amide Int03 through condensation with *N,N*-dimethylformamide dimethyl acetal (DMF-DMA) and cyclization of the intermediate active imine with hydrazine (Xu et al., 2010). The *N*-acylsulfonamide BA-26 was prepared by CDI-mediated acylation of methane-sulfonamide with bumetanide.

#### 2.1.3 Preparation of sulfoximines

Derivatives of sulfoximines have been elaborated from the commercially available 4-chloro-3-(chlorosulfonyl)-benzoic acid (Scheme 2). Nitration followed by the reduction of the chlorosulfonyl intermediate using triphenylphosphine as the reducing agent (Buckler et al., 1962) produces the intermediate Int09. After concomitant methylation of thiophenol and carboxylic acid, sulfoximine was generated through phenyliodonium diacetate (PIDA)-mediated oxidation (Bull et al., 2017). The nitro group was reduced to aniline using hydrogen and the Pd/C catalyst. Alkylation under reductive amination conditions with butyraldehyde produces the desired product together with side products that were easily separated by flash chromatography. The final hydrolysis under basic conditions led to the desired sulfoximine BA-33.

An improved strategy was developed for the preparation of the *N*-methyl-sulfoximine analog BA-134. The *N*H-sulfoximine Int11 described above was methylated by applying Eschweiler–Clarke conditions (Clarke et al., 1932; Eschweiler, 1905). Iron-mediated nitro reduction delivered the aniline that was cleanly alkylated using classical reductive amination conditions. The targeted carboxylic acid BA-134 was obtained after lithine-mediated saponification.

**SCHEME 3:**
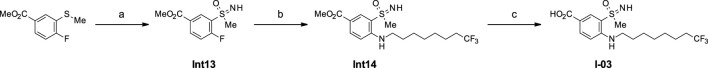
Synthesis of the sulfoximine I-03 compound. Reagents and conditions: **(a)** PIDA, H_2_NCO_2_NH_4_, MeOH, 20 °C; **(b)** H_2_N-(CH_2_)_7_-CF_3_, dioxane, 110 °C; **(c)** NaOH, THF, 20 °C.

Compound I-03 was prepared as described in Scheme 3. Commercially available 4-fluoro-3-methylthio-benzoate was converted into sulfoximine through PIDA oxidation. SN_Ar_ using trifluorooctylamine followed by saponification with lithine produced the desired product I-03.

#### 2.1.4 Preparation of sulfonimidamides

**SCHEME 4:**
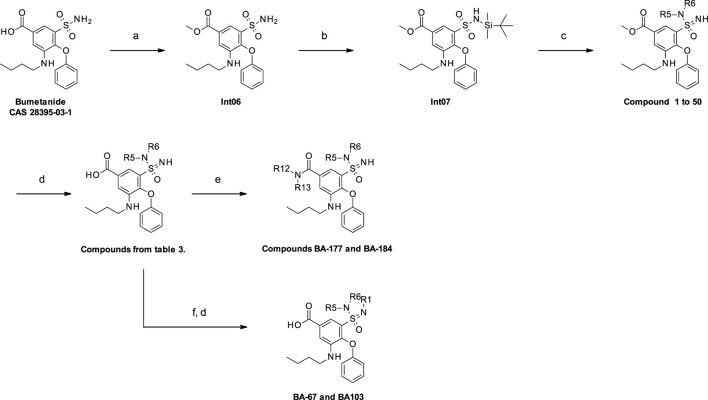
Synthesis of sulfonimidamides. Reagents and conditions: **(a)** H_2_SO_4_, MeOH, 65 °C; **(b)** TBDMSCl, Et_3_N, THF, 20 °C; **(c)** i. PPh_3_, C_2_Cl_6_, CHCl_3_, 70 °C; ii. Et_3_N, CHCl_3_, 0 °C; iii. R_5_R_6_NH, 0 °C to 20 °C; iv. HCl, MeCN, 20 °C or TBAF 1 M in THF, CH_3_CN, 20 °C; **(d)** LiOH, THF/H_2_O/MeOH (1/1/1), 20 °C or Me_3_SiOK, THF, 20 °C; **(e)** R_12_R_13_NH, HATU, DMF, 20 °C; **(f)** R_1_B(OH)_2_, Cu(OAc)_2_, pyridine, dioxane, 100 °C.

Sulfonimidamides were prepared following the procedure developed by Chen and Gibson (2015) (Scheme 4). Silylated bumetanide methyl ester was subjected to triphenyldichlorophosphorane (Ph_3_PCl_2_) to produce sulfonimidoyl chloride. This intermediate was *in situ* substituted with a set of diverse amines and anilines to obtain silyl-protected sulfonimidamides. These were ultimately deprotected by acidic workup or by treatment with TBAF. Lithine-mediated ester hydrolysis delivered the target compounds in the pure form. Notably, the unsubstituted sulfonimidamides (R1 = R5 = R6 = H) could not be prepared by this route as we observed degradation during the silyl group cleavage using TBAF.

#### 2.1.5 Preparation of sulfonamides

**SCHEME 5:**
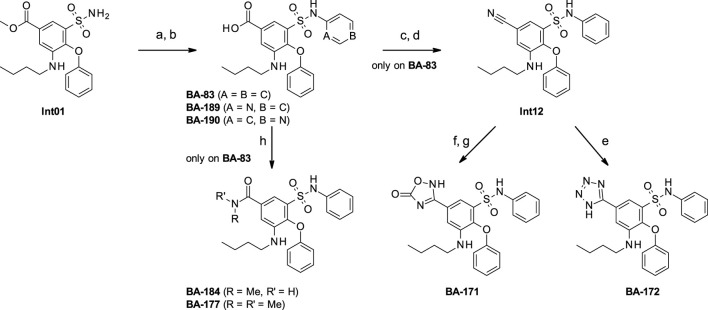
Synthesis of sulfonamides. Reagents and conditions: **(a)** PhBr, CuI, N,N′-dimethylethylenediamine, K_2_CO_3_, CH_3_CN, 80 °C; **(b)** LiOH, THF/H_2_O/MeOH (1/1/1), 20 °C; **(c)** i. CDI; CH_3_CN, 50 °C; ii. NH_4_OH, 20 °C; **(d)** Pyr., TFAA, dioxane, 20 °C; **(e)** NaN_3_, NH_4_Cl, DMF, 120 °C; **(f)** NH_2_OH.HCl, NaHCO_3_, EtOH, 80 °C; **(g)** CDI, DBU, dioxane, 100 °C; **(h)** RR’NH, HATU, DIPEA, DMF, 20 °C.

Sulfonamides were synthesized via copper-mediated cross-coupling on the intermediate Int01 following the protocol established by the Buchwald group [Scheme 5 (Klapars et al., 2001)]. Subsequent lithine-mediated saponification yielded compounds BA-83, BA-189, and BA-190. Peptide coupling reaction on BA-83 produced BA-177 and BA-184. The carboxylic acid moiety of BA-83 was also transformed into the corresponding nitrile Int15 through amide dehydration using TFAA. Applying the same synthetic route as for BA-06 on the nitrile Int15 led to the formation of BA-171. Finally, treatment of the nitrile with sodium azide successfully yielded the tetrazole derivative BA-172.

### 2.2 Determination of NKCC1 function

**FIGURE 3:**
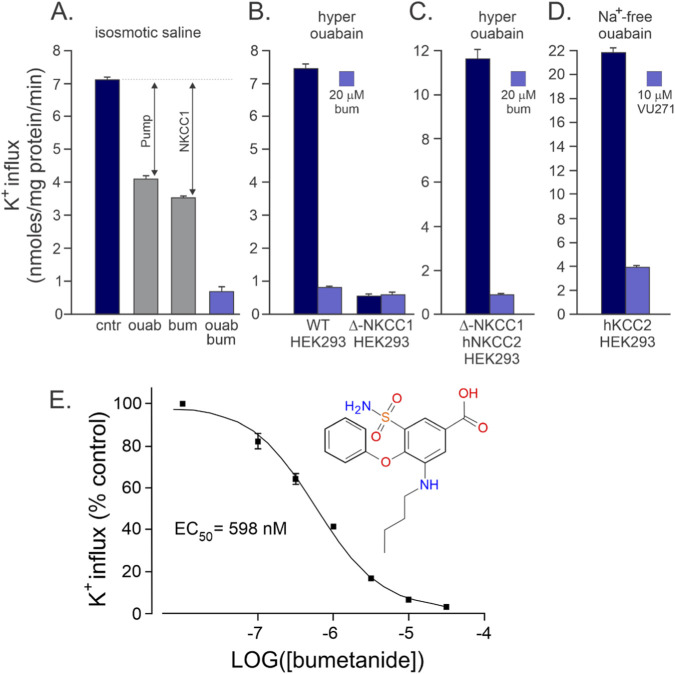
Unidirectional K^+^ influx measured in HEK293 cells. **(A)** Flux measured in the absence of drugs, in the presence of 100 μM ouabain, in the presence of 20 µM bumetanide, and in the presence of both drugs. **(B)** Flux measured under hypertonic (370 mOsM) conditions in the presence of 100 µM ouabain. Wild-type cells exhibit a bumetanide-sensitive large K^+^ influx, whereas NKCC1-knockout cells show minimal flux, which is not responsive to bumetanide. **(C)** K^+^ flux measured in HEK293 cells lacking NKCC1 (△NKCC1) and stably transfected with hNKCC2. K^+^ influx was measured in the presence and absence of 20 µM bumetanide. **(D)** K^+^ influx measured in HEK293 cells overexpressing hKCC2 (Prael Iii et al., 2022). The flux was measured in the presence or absence of 10 µM of the K–Cl cotransporter-specific inhibitor, VU0463271. **(E)** Dose–response curve of bumetanide on unidirectional ouabain-resistant K^+^ influx. The concentrations tested ranged from 100 nM–31.6 μM. Cntr = control, ouab = ouabain, bum = bumetanide, VU271 = VU0463271.

To assess the activity of compounds on NKCC1 function, K^+^ influx measurements were performed in HEK293 cells exposed to hypertonicity. As shown previously (Magaña-Ávila et al., 2025), bumetanide-sensitive K^+^ influx in HEK293 cells is significantly enhanced under hypertonic conditions compared to that in isotonic conditions. K^+^ influx was measured using radioactive ^83^Rb as a tracer. The flux was conducted in the presence of 100 µM ouabain to prevent uptake through the Na^+^/K^+^ pump. Under these conditions, most of the flux is mediated by NKCC1 (Figure 3A). In each experiment, the K^+^ influx measured in the absence of drugs subtracted from the K^+^ influx measured in the presence of 20 µM bumetanide represented the total NKCC1-mediated flux. The inhibition of K^+^ flux by each drug (tested at 2 µM or 20 µM) was compared to the total flux inhibition observed in the presence of bumetanide. Bumetanide, furosemide, and ARN23746 were used as references in the assay. Notably, some compounds at 20 μM yielded flux values that were slightly lower than those obtained with 20 μM bumetanide, indicating stronger inhibition. This led to percentage values that were slightly higher than 100%.

### 2.3 Structure–activity relationship of the analogs

SAR exploration started from the R1 carboxylic acidic part replacement (Table 1). Triazolone- and oxadiazolone-containing compounds (BA-05, BA-06, and BA-24) maintain a moderate level of activity. Despite a relatively acidic hydrogen atom, the *N*-acylsulfonamide compound BA-26 shows only very weak inhibition, which is possibly attributed to the steric hindrance caused by the bulky methane-sulfonamide group. Interestingly, compounds featuring a heteroaryl group without any acidic proton (BA-01 and BA-13) still retain a moderate level of inhibition of NKCC1.

**TABLE 1:** Inhibitory activity of R1-modified derivatives. The percentage of NKCC1 inhibition was measured at both 2 μM and 20 μM. Indicated values are compared to inhibition by 20 μM bumetanide, set at 100%.

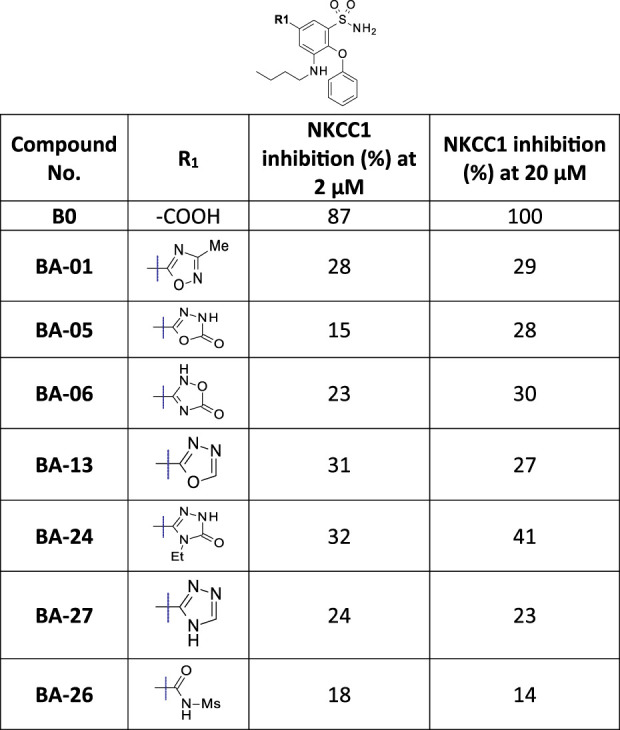

We then prepared a small set of sulfoximines and sulfonimidamides derivatives substituted with a methyl substituent (Table 2). At 20 µM, sulfoximines BA-33 and BA-175 show a loss of inhibition compared to bumetanide (B0), although at 2 µM, only BA-175 retains a significant level of inhibition. In the sulfonimidamide series, derivative BA-49 leads to a similar inhibition compared to that with sulfoximines, while mono-substituted derivative BA-50 shows the highest inhibition for the series (79% at 20 µM and 55% at 2 µM). Derivative I03, a sulfoximine based on the scaffold of ARN23746, was nearly inactive.

**TABLE 2:** Inhibitory activity of substituted sulfonamides, sulfoximines, and sulfonimidamides. The percentage of NKCC1 inhibition was measured at both 2 μM and 20 μM. Indicated values are compared to inhibition by 20 μM bumetanide, set at 100%.

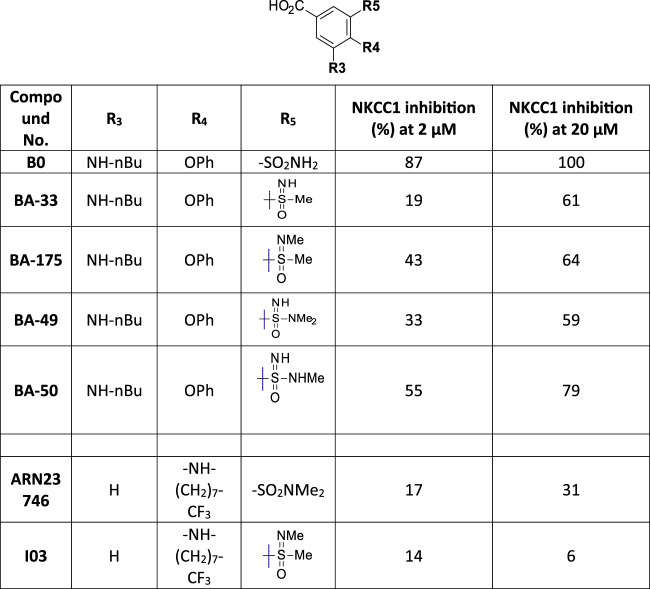

Based on the promising activity of BA-50, several sulfonimidamides analogs were further prepared, with up to three substituents on the sulfonimidamide nitrogens, including a diverse range of alkyls, aryls, and hetero-aryls substituents (Table 3).

**TABLE 3:** Inhibitory activity of mono- and di-substituted sulfonimidamides. The percentage of NKCC1 inhibition was measured at both 2 μM and 20 μM. Indicated values are compared to inhibition by 20 μM bumetanide, set at 100%.

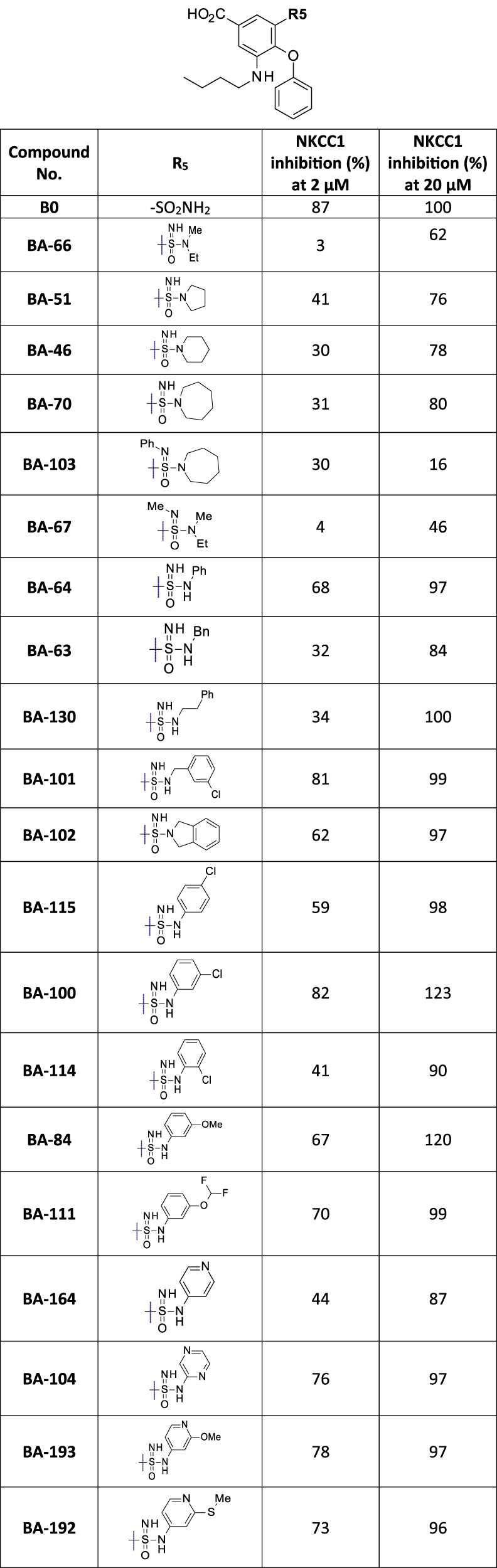

The inhibition of NKCC1 appeared to be highly influenced by the size of the alkyl residues (see, for example, the increased inhibition observed for BA-66, BA-51, BA-46, and BA-70). We were also pleased to observe a strong inhibition for the phenyl-substituted analog BA-64. Compounds BA-130 and BA-63 further highlight the positive effect of having an aromatic ring correctly positioned at this particular position. The comparison of compounds BA-102 and BA-51 further emphasizes this aromatic effect. When comparing compounds BA-66, BA-67, BA-103, and BA-70, a pattern emerged, namely, maintaining a hydrogen bond donor on the sulfonimidamide group is crucial for retaining a high NKCC1 inhibitory activity. The substitution of the aryl ring was further analyzed, and the meta substitution appeared to be the most favored one (see compounds BA-111 and BA-84), while the electronic nature of the substituent did not appear to significantly impact the inhibition (for example, compare compounds BA-84, bearing a methoxy group, and BA-100, bearing a chlorine). The introduction of a heterocycle ring (pyridine and pyrazine) was tolerated, and further substitution was also possible (compounds BA-164, BA-193, BA-192, and BA-104). Importantly, at a concentration of 20 µM, compounds BA-100 and BA-84 demonstrated greater inhibition than bumetanide.

As aryl-substituted derivatives caused unanticipatedly high inhibition, we decided to further explore this effect by preparing aryl-substituted sulfonamide derivatives. The activity profile confirms that an aryl substitution leads to an increased inhibition of NKCC1; although in this case, the introduction of a pyridine was surprisingly less active (Table 4).

**TABLE 4:** Inhibitory activity of substituted sulfonamides. The percentage of NKCC1 inhibition was determined at both 2 μM and 20 μM. Indicated values are compared to inhibition by 20 μM bumetanide, set at 100%.

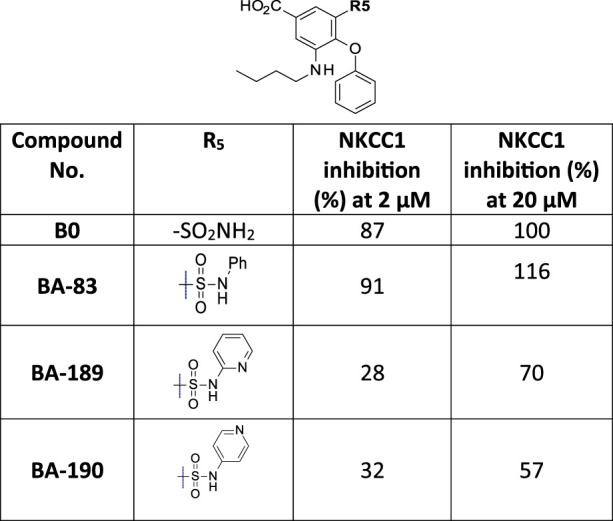

Having found good substituents for the R5 position, we decided to reanalyze the R1 position to modulate the physico-chemical parameters through the tuning of the acidic proton at this position. Unfortunately, as previously observed, a strong reduction in inhibition was observed when introducing an oxadiazolone residue. Despite this lowered affinity, compound BA-172, bearing a tetrazole bioisoster for the carboxylic acid, remains highly active at 20 µM. Attempts to replace the carboxylic acid with various amides, for example, in compounds BA-177 and BA-184, led to a complete loss of inhibition (Table 5).

**TABLE 5:** Inhibitory activity of substituted sulfonamides with modifications of R1. The percentage of NKCC1 inhibition was determined at both 2 μM and 20 μM. Indicated values are compared to inhibition by 20 μM bumetanide, set at 100%.

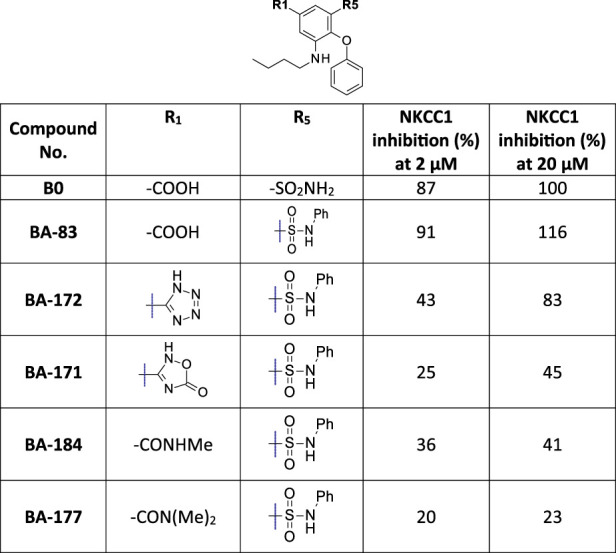

**FIGURE 4:**
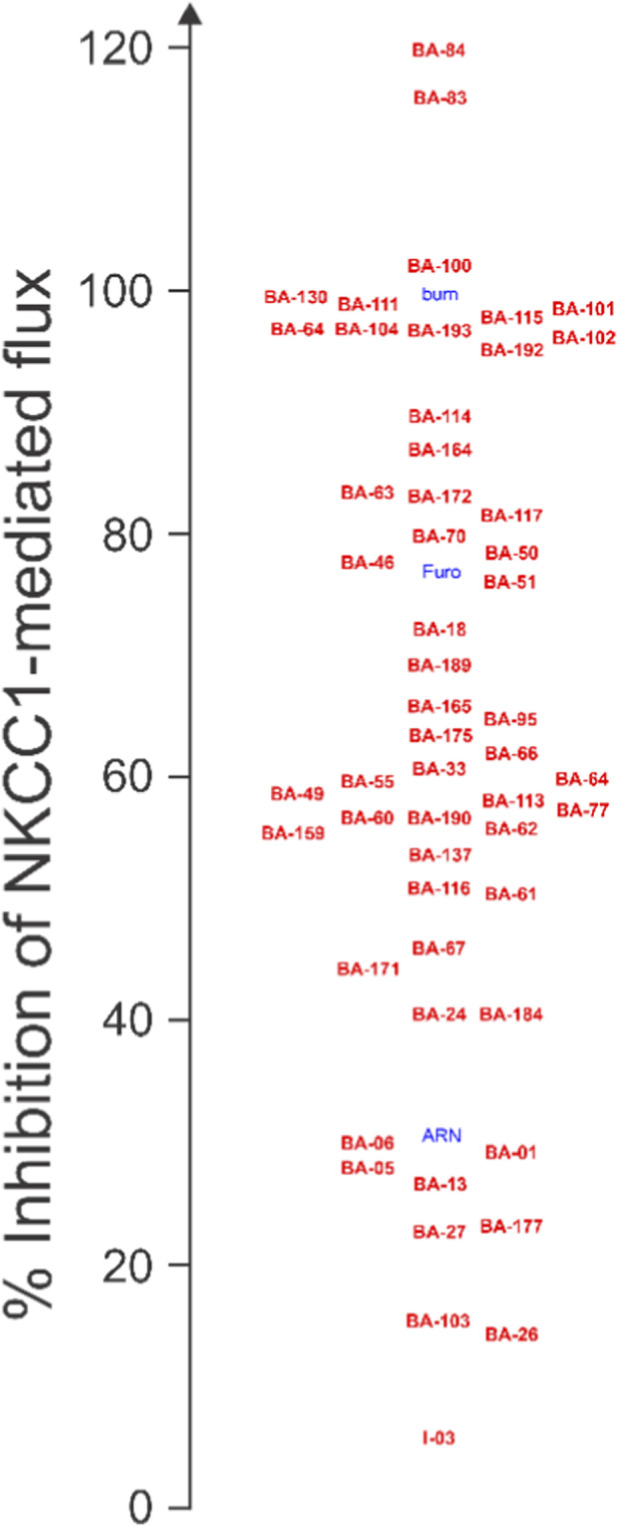
Summary of the inhibitory activity of 51 compounds. The percentage of inhibition at 20 μM is illustrated for the 51 compounds (in red) described in this study, relative to bumetanide set at 100%. The inhibitory activity of bumetanide, furosemide, and ARN23746 as references is indicated in blue.

A summary of all the NKCC1 inhibition data is provided in graphical form in Figure 4.

### 2.4 Structural features revealed by docking experiments

**FIGURE 5:**
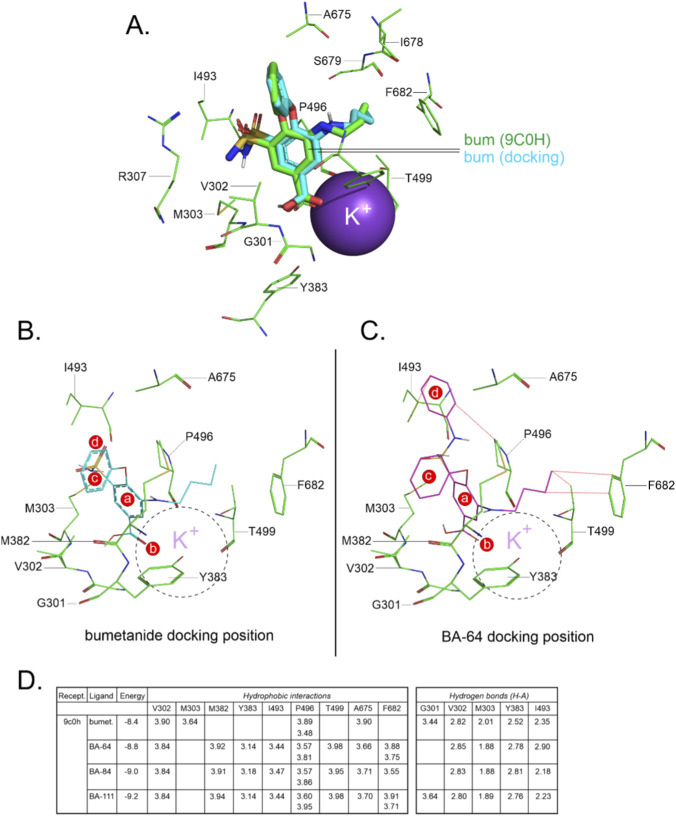
Docking of ligands to the NKCC1 structure. The NKCC1 structure used was pdb: 9c0h. **(A)** Superimposition of bumetanide docking generated by AutoDock Vina (blue) versus bumetanide in 9c0h structure (green). Similar poses of bumetanide **(B)** and BA-64 **(C)** with NKCC1 residues surrounding the inhibitor molecules that are labeled. The position of the K^+^ ion is indicated by a dashed circle. Red circles highlight key features of the molecules: a, the central phenyl ring; b, the carboxyl group; c, R4; and d, R5. BA-64 forms new interactions (red dash lines) with residues I493, T499, and F682. The best position for each ligand was captured as a *pdbqt* file and converted into the *pdb* format using OpenBabel. **(D)** Table summarizing the receptor–ligand interactions. Receptor and ligand were combined with pymol to create a new *pdb* file that was uploaded into PLIP (Protein Ligand Interaction Profiler, Biotechnology Center, TU Dresden) to identify non-covalent interactions. Docking energies, expressed in kcal/mol, are given in column 3. Hydrophobic interactions are provided as distances to specific amino acid residues in columns 4–13. Hydrogen bonds are provided as H–A distances in columns 14–20. Distances are measured in Å.

Docking of compounds has been achieved to explain the strong inhibition of compounds having an aryl substituent at the R5 position. The cryo-EM structure (pdb: 9c0h) was used for docking. The center coordinates of the bumetanide molecule were extracted from the 9c0h structure, and the inhibitor was deleted before creating a new pdb file. Using the new file and the extracted coordinates as a starting point, bumetanide and various compounds were re-docked independently. We confirmed that the docking software placed bumetanide in a pose that is very similar to that of the original structure (Figure 5A). As shown in Figures 5B,C, BA-64 produces a pause that overlaps well with the structure of bumetanide. The central phenyl ring (red dot a) and the carboxyl group (red dot b) have similar poses, allowing the side chain of Tyr383, which is a key residue in the binding of K^+^ (Chew et al., 2019; Liu et al., 2019; Moseng et al., 2022; Zhao et al., 2025), to interact and form hydrogen bonds with bumetanide. While the aromatic group R4 (red dot c) has a slightly different pose than bumetanide, additional interactions are noted within a lipophilic region, particularly at the isoleucine residue 493, where the aromatic group is positioned (red dashed line). In addition,notably, the ring in R5 (red dot d) is unencumbered and forms a new hydrophobic interaction with M382 (Figure 5B). These new interactions likely maintain the binding of BA-64 to the cotransporter (inhibitory strength similar that of bumetanide) despite the loss of other points of contact. Notably, the given docking energies of BA-64, BA-83, and BA-111 were slightly higher than that of bumetanide. However, poor correlation was observed between the docking energy of the compounds and their inhibitory activity at either 2 μM or 20 μM (r^2 = 0.423, measured with over 30 compounds), indicating that the overall docking energy value is a poor predictor of the actual inhibitory strength determined in functional assays.

### 2.5 Further characterization of the select compounds

The EC_50_ for NKCC1 inhibition was measured for the more potent inhibitors, and the kinetic solubility was also evaluated (Table 6). Compounds derived from bumetanide with phenyl sulfonamide and aryl sulfonimidamide groups are found to be more potent than bumetanide, while maintaining good solubility. In particular, compound BA-83 shows a good profile with an EC_50_ of 150 nM. In addition, NKCC2 and KCC2 inhibition was measured at two concentrations (2 and 20 µM). The validation of NKCC2 and KCC2 fluxes are provided in Figures 3B–D. The results indicated that at 20 µM, all the tested compounds retain a good inhibition for NKCC2 but not for KCC2. At a lower dose (2 µM), NKCC2 inhibition was significantly lowered, indicating that the potent compounds developed in this study show a good selectivity for NKCC1 over NKCC2 and KCC2. The solubilities of several newly synthesized compounds were measured and found to be similar or slightly better than that of bumetanide.

**TABLE 6:** Selectivity profile and solubility of selected NKCC1 inhibitors. EC_50_ was measured using a 6.

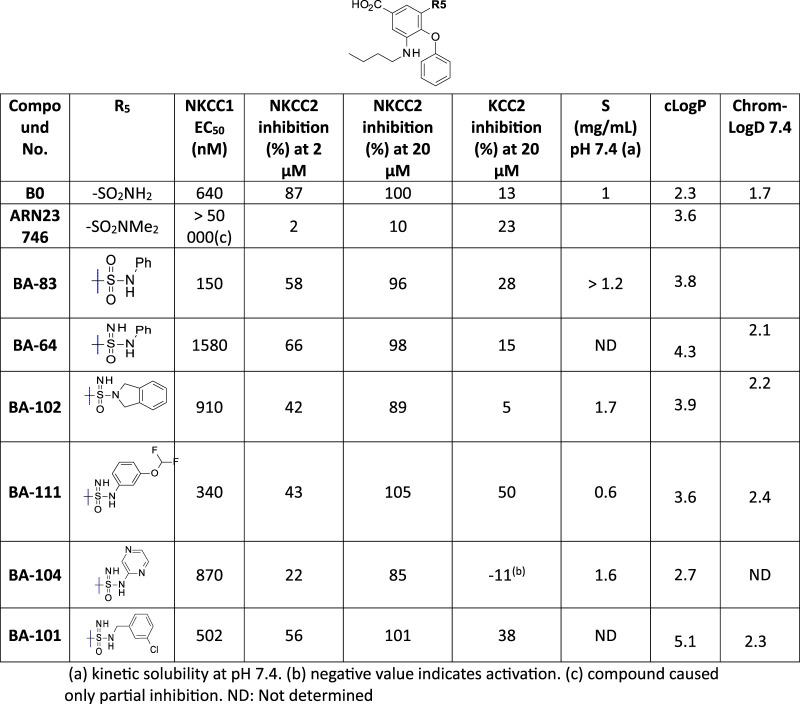

## 3 Discussion

### 3.1 NKCC1 inhibitors are promising putative drugs to treat a wide range of disorders

NKCC1 is at the core of extensive research to synthesize novel inhibitors as potential therapeutic perspectives. Hyperactivity of this cotransporter is observed in many disorders, notably some that are orphan diseases. The regulation of ions, and notably chloride, is deficient following hyperactivity episodes and inflammatory signals, among others. In addition, NKCC1 activity affects the tight junctions, thereby also acting on the inflammatory signals (Koumangoye et al., 2022; Koumangoye et al., 2026). Agents that are capable of restoring the physiological levels of these intracellular ions by blocking NKCC1 constitute promising targets. Bumetanide is the prototype that has been extensively studied in many animal models and clinical trials. Here, we examined two out of four structural substitution sites of benzene-sulfonamide that can be modified using multiple chemical approaches. In particular, they are sulfoximines and sulfoximidamides that have never been tested before to the best of our knowledge. Many inhibitors were synthesized with a large degree of structural diversity and biological efficiency.

We have developed a synthetic route to prepare a series of analogs bearing modifications at the R1 and R5 positions. We discovered unexpected NKCC1 inhibition with compounds having an *N*-phenyl substitution on sulphonamides and sulfonimidamides. The best derivatives, which have this aromatic substitution at R5, have shown sub-micromolar NKCC1 inhibition, good selectivity over related targets (NKCC2 and KCC2), and good solubilities. Replacement of the carboxylic acid at position R1 remains a challenge, leading to a substantial decrease in activity. Further work is underway to evaluate the ADME properties and preclinical efficacy of the new inhibitors.

Many of the compounds identified have similar efficacy to inhibit NKCC1 as bumetanide, and some are better than the latter; to the best of our knowledge, this is unprecedented. This is in sharp contrast to the lower affinity measured for ARN23746 (this study) and reported by Savardi et al. (2020). Overall, when tested on NKCC2 function, all compounds active for NKCC1 were also active for NKCC2, although at slightly lower efficacy. This was also the case for ARN23746. Indeed, the compound was less efficacious on NKCC2 than on NKCC1 in our study as well. As explained in the methods section, we were particularly careful in setting up our NKCC2 assay as we believe that it cannot be carried out in cell lines that express native NKCC1, hence the need to test the molecules on NKCC2-transfected HEK293 cells that are deficient in NKCC1. Furthermore, we were also conscientious in validating the functionality of the human NKCC2 clone obtained from OriGene. We demonstrated that the OriGene clone used by Savardi et al. (2020) was non-functional due to the presence of an epitope tag at the extreme carboxyl-terminus. Once we removed the extra amino acids and restored the original carboxyl-terminal tail, we recovered its functionality. We also identified mutations located in exon 5 in the OriGene clone. The sequence did not match the sequence of the three possible exons 5 (exons 5A, 5B, and 5F) identified in the human genome. We then substituted the mutated sequence with exon 5A of the human gene. Thus, we cannot confirm that the NKCC2 data of Savardi were not in fact mediated by NKCC1, as the NKCC2 clone, if used as purchased, was non-functional. Irrespective of whether the compounds act similarly on NKCC2 versus NKCC1 or not, the overall properties of the compounds are important, and their pharmacokinetics properties will determine whether they will cause diuresis and the accompanying loss of K^+^ associated with the inhibition of NKCC2.

Our docking experiments confirm that bumetanide has a pose that is comparable to that of the loop diuretic in PDBs, namely, 9c0h and 7xs1. Residues involved in binding coordination are Gly301, Val302, Met303, Tyr383, and Ile493 forming hydrogen bonds with bumetanide and Gly301, Val302, M303, Tyr383, and Ile493 forming hydrophobic interactions with the inhibitor. Binding of the novel compounds typically involves the same residues, with a few additional hydrophobic interactions. The additional neighboring residues that coordinate the interaction are Met382, Thr499, and Phe682. The shorter distance interactions for the residue Met303 and the evidence of additional hydrophobic interactions with Met382, Tyr383, I493, T499, and Phe282 might explain the greater inhibitory effect of BA-84 than bumetanide. We found that the docking energies provided by the docking software are overall poorly correlated with the inhibitory strength of the compounds. This is likely due to the intrinsic limitations of molecular docking; for instance, the reliance on a unique rigid protein structure captured by cryo-EM instead of a highly dynamic protein that likely exists in the plasma membrane under physiological conditions.

### 3.2 The ubiquitous presence of NKCC1 and the blood–brain barrier issues

It has been repeatedly proposed that NKCC1 inhibitors are not good candidates to treat disorders because of the presence of NKCC1 in most cells. This argument is, however, circular and incompatible with the wide range of inflammatory agents (aspirin, paracetamol, or X other agents) that act on a multitude of central and peripheral targets. The presence of a target in many/most different types of cells does not imply that targeting this site will fall short of treating disorders; cell swelling due to changes of ionic gradients and water influx is common in many insults, and rejecting putative agents on this basis cannot be sustained from a medical standpoint. In addition, the unprecedented scope of peripheral and central disorders in which NKCC1 is hyperactive, KCC2 is hypoactive, and NKCC1 inhibition (or KO in animal models) attenuates the disorders justifies our efforts to synthetize novel NKCC1 inhibitors to test them in cognitive aims and possibly therapeutic perspectives to treat pharmaco-resistant peripheral or central disorders in the future.

Yet, we would like to stress the following points concerning the BBB permeability issue despite its limited relevance to the present study. It bears stressing that there is no single reliable measure of BBB bumetanide permeability in humans and *a fortiori* patients with epilepsies, autism, or brain tumors. Measures of BBB permeability and drug distribution in rodents cannot be extrapolated to humans as there are major differences, including the types of neurons, biochemical processes, and lifetime of drugs (the effect of bumetanide is more prolonged in humans than in rodents), along with the large number of disorders in which the agents are efficacious in rodents but not in humans. This is not to state that bumetanide or our analogs do cross the BBB but to propose that caution ought to be exerted in this domain. The success of clinical trials to attenuate syndromes in temporal lobe epilepsy, infantile epilepsies, and autism cannot be readily reconciled with this restricted and over-publicized view, and this has already been debated elsewhere (Delpire and Ben-Ari, 2022; Hadjikhani et al., 2015; Hadjikhani et al., 2018; Lemonnier et al., 2017). From a medical perspective, the BBB permeability is not central because if the drug achieves an efficient action even *via* a peripheral mechanism, the treatment remains valid. Over 1,030 children with autism have been treated successfully in seven phase-2 trials performed in five different countries (Xiao et al., 2024), and more recently, a worldwide phase-3 trial failed; however, using machine learning, we have succeessfully identified over 30%–40% of children within the failed phase-3 trial that responded to the treatment (Rabiei et al., 2026). This is in accord with the now well-established need to subdivide populations of patients with highly heterogeneous disorders such as autism or fragile X syndrome because a single treatment to treat such heterogeneous disorders is bound to fail. Here, we propose a series of novel molecules that will most likely be found to act on other targets as well, including peripheral targets, thereby enhancing the range of tests possible first in animal models and then in pre-IND tests and clinical trials for the best of them.

In conclusion, we have identified a novel chemical class of selective NKCC1 inhibitors that could serve as probes for proof-of-concept and for further identification of clinical candidates for treating various types of cancer and NKCC1-related disorders.

## 4 Materials and methods

### 4.1 Chemical synthesis

#### 4.1.1 General information

^1^H and ^13^C NMR spectra were recorded on a BRUKER DPX 200 or a BRUKER Advance 400 spectrometer. The coupling constants J are reported in Hertz (Hz). Multiplicity is indicated as follows: s (singlet), d (doublet), t (triplet), q (quartet), sext (sextuplet), sept (septuplet), oct (octuplet), dd (doublet of doublet), bs (broad singlet), and m (multiplet). All air- and moisture-sensitive manipulations were performed under an argon atmosphere with anhydrous solvents in flame-dried glassware. Commercially available reagents were used without further purification, unless otherwise indicated. Analytical TLC was performed using Merk precoated silica gel 60 F-254 sheets with spot detection under UV light or using potassium permanganate stain or cerium ammonium molybdate stain. Flash column chromatographies were carried out using amorphous Phenomenex Flash Silica (CS); particle size of 40 µm–60 µm; 60 A on a Biotage Isolera system.

#### 4.1.2 Sulfonimidamide preparation, general procedure 1 (GP1)—method A

A solution of triphenylphosphine (1.1 equiv.) and hexachloroethane (1.1 equiv.) in CHCl_3_ (0.6 M) was stirred at 70 °C for 3 h. After cooling down to 20 °C, triethylamine (1.5 equiv.) was added to the white suspension. The resulting yellow suspension was stirred for 10 min at 20 °C and was cooled down to 0 °C. A solution of **Int07** (1 equiv.) in CHCl_3_ (0.6 M) was added, and the resulting clear solution was stirred for 20 min at 0 °C. Amine (3 equiv.) in CHCl_3_ (2 M) was added, and the resulting solution was heated up to 20 °C and stirred for 2 h. The solvent was removed, and the crude mixture was diluted in CH_3_CN (0.1 M). HCl 37% (0.5 v/v CH_3_CN) was added at 20 °C, and the resulting solution was stirred at 20 °C for 45 min. The solution was basified to pH 10 using a saturated solution of NaHCO_3,_ and the aqueous layer was extracted with EtOAc. The combined organic layers were dried over Na_2_SO_4_, filtered, and concentrated under reduced pressure to obtain the crude sulfonimidamide. The crude residue was purified by automated flash chromatography with cyclohexane/EtOAc (gradient from 1/0 to 0/1 over 10 CV) to obtain the pure compound.

#### 4.1.3 Sulfonimidamide preparation—method B

A solution of triphenylphosphine (1.1 equiv.) and hexachloroethane (1.1 equiv.) in CHCl_3_ (0.6 M) was stirred at 70 °C for 3 h. After cooling down to 20 °C, triethylamine (1.5 equiv.) was added to the white suspension. The resulting yellow suspension was stirred for 10 min at 20 °C and was cooled down to 0 °C. A solution of **Int02** (1 equiv.) in CHCl_3_ (0.6 M) was added, and the resulting clear solution was stirred for 20 min at 0 °C. Amine (3 equiv.) in CHCl_3_ (2 M) was added, and the resulting solution was heated up to 20 °C and stirred for 2 h. The solvent was removed, and the crude mixture was diluted in CH_3_CN (0.1 M). A solution of tetrabutylammonium fluoride (1M in THF, 1 equiv.) was added at 20 °C, and the resulting solution was stirred at 20 °C for 5 h. If necessary, a solution of tetrabutylammonium fluoride (1M in THF, 2 equiv.) was added at 20 °C, and the resulting solution was stirred at 20 °C until full conversion was observed (conversion monitored by LC–MS). Water was added to the reaction mixture that was extracted with a mixture of CHCl_3_: iPrOH (8:2). The combined organic layer was concentrated under reduced pressure. The crude residue was purified by automated flash chromatography with cyclohexane/EtOAc (gradient from 1/0 to 0/1 over 10 CV) to obtain the pure compound.

#### 4.1.4 Methyl ester saponification, general procedure 2 (GP2)—method A

LiOH (2 equiv.) was added to a solution of methyl ester (1 equiv.) diluted in a mixture of THF/H_2_O/MeOH 1/1/1 (0.1 M). The resulting mixture was stirred for 2 h at 20 °C or until full conversion was observed. THF was removed, and the mixture was diluted in water (5 mL). The aqueous layer was washed with EtOAc and was acidified with HCl 1N until pH 2–3. The aqueous layer was extracted with EtOAc, and the resulting organic layer was dried over Na_2_SO_4_, filtered, and concentrated under reduced pressure to obtain the corresponding carboxylic acid.

#### 4.1.5 Methyl ester saponification, general procedure 2 (GP2)—method B

In a sealed tube, potassium trimethylsilanolate (2.4 equiv.) was added to a solution of methyl ester (1 equiv.) diluted in dry tetrahydrofuran (0.2 M). The reaction was stirred at 20 °C for 16 h. Portions of potassium trimethylsilanolate (0.6 equiv.) could be added every 4 h to complete conversion (conversion monitored by LC–MS). Once the full conversion was reached, water was added to the reaction mixture. The aqueous layer was acidified upon the addition of an aqueous solution of HCl 1N to reach pH 2–3. The organic layer was extracted thrice with DCM, and the combined organic layers were washed oncd with brine, dried over MgSO4, filtered, and concentrated under reduced pressure to obtain the corresponding carboxylic acid.

#### 4.1.6 Peptide coupling reaction, general procedure 3 (GP3)

DIPEA (1.5 equiv.) and HATU (1.2 equiv.) were added to a solution of carboxylic acid (1 equiv.) in N,N-dimethylformamide (0.1 M) at 20 °C. The resulting mixture was stirred at 20 °C till complete conversion (conversion monitored by LC–MS). The resulting solution was poured into a saturated aqueous solution of NH_4_Cl and extracted twice with DCM. The combined organic layers were washed with brine, dried over MgSO4, filtered, and concentrated under reduced pressure. The crude compound was purified by reverse-phase LC–MS. The pure fractions containing the target compounds were collected and concentrated under reduced pressure to provide pure compounds.

#### 4.1.7 Sulfonamide arylation, general procedure 4 (GP4)

Potassium carbonate (2.5 equiv.), copper(I) iodide (0.1 equiv.), and N,N′-dimethylethylenediamine (0.5 equiv.) were added to a stirred solution of **Int01** (1 equiv.) in dry acetonitrile (0.2 M). The solution was degassed under argon and sonication, and then ArylBromide (1.20 equiv.) was added at 20 °C. The reaction vessel was flushed with argon, sealed, and stirred at 80 °C for 16 h. The reaction was cooled down to 20 °C and filtered over a pad of celite to obtain a crude compound that was purified by automated flash chromatography with cyclohexane/EtOAc (gradient from 100/0 to 0/100 over 10 CV) or with DCM/MeOH (gradient from 100/0 to 80/20 over 10 CV) to obtain the corresponding aryl-substituted sulfonamide.

#### 4.1.8 Compound BA-01

Chemical structure diagram of a sulfonamide compound displaying a central benzene ring bonded to a sulfonamide group, a phenyl group with an oxygen linker, and a substituted imidazole ring with a nitrogen-containing side chain.

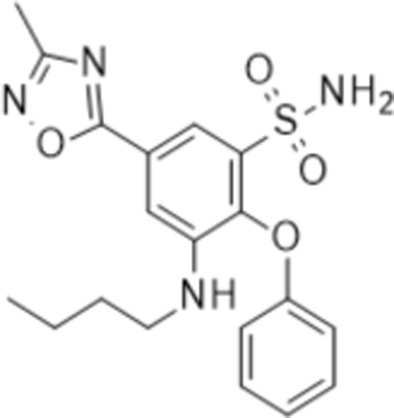

3-(butylamino)-5-(3-methyl-1,2,4-oxadiazol-5-yl)-2-phenoxy-benzenesulfonamide (**BA-01**).

Carbonyl diimidazole (49 mg, 0.301 mmol, 1.1 equiv.) was added to a stirred solution of **Bumetanide** (100 mg, 0.274 mmol, 1 equiv.) in acetonitrile (2.7 mL, 0.1 M), and the solution was stirred at 50 °C for 1 h; then, N-hydroxyacetamidine (23 mg, 0.315 mmol, 1.15 equiv.) was added, and the reaction mixture was sirred at 20 °C for 16 h. The reaction mixture was concentrated under reduced pressure, and the crude oil was taken up in pyridine (2 mL) and stirred at 110 °C for 16 h. The reaction mixture was concentrated under reduced pressure and purified by automated flash chromatography with DCM/MeOH (gradient from 1/0 to 95/5 over 10 CV) to obtain **compound BA-01** (30 mg, 0.071 mmol, 26%) as a white solid.

C19H24N4O5S; MS (ESI+) m/z: 403 [M + H]+; 1H NMR (400 MHz, DMSO-d6): δ 7.78 (d, J = 2.0 Hz, 1H), 7.55–7.42 (m, 3H), 7.28 (dd, J = 8.7, 7.3 Hz, 2H), 7.09–6.98 (m, 1H), 6.93–6.84 (m, 2H), 5.30 (t, J = 5.7 Hz, 1H), 3.12 (q, J = 6.6 Hz, 2H), 2.45 (s, 3H), 1.49–1.33 (m, 2H), 1.21–1.05 (m, 2H), and 0.79 (t, J = 7.3 Hz, 3H).

#### 4.1.9 Compound BA-13

Chemical structure diagram showing a sulfonamide compound with a benzene ring core, triazole ring, sulfonamide group, substituted aniline, phenyl ether, and pentyl side chain branching from the central ring.

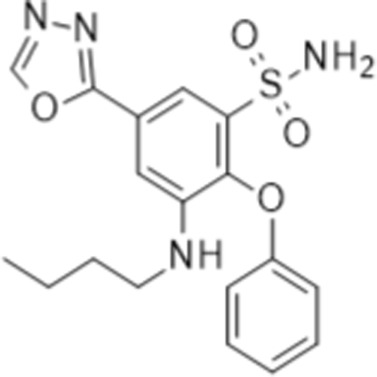

3-(butylamino)-5-(1,3,4-oxadiazol-2-yl)-2-phenoxy-benzenesulfonamide (**compound BA-13**).

(N-Isocyanoimino)triphenylphosphorane (199 mg, 0.658 mmol, 1.2 equiv.) was added to a stirred solution of **Bumetanide** (200 mg, 0.548 mmol, 1 equiv.) in 1,2-dichloroethane (5.5 mL, 0.1 M), and the solution was stirred at 20 °C for 16 h. The reaction mixture was concentrated under reduced pressure and triturated in EtOAc to obtain **compound BA-13** (18 mg, 0.046 mmol, 8%) as a white solid.

C18H20N4O4S; MS (ESI+) m/z: 389 [M + H]+; 1H NMR (400 MHz, DMSO-d6): δ 9.38 (s, 1H), 7.72 (d, J = 2.0 Hz, 1H), 7.51–7.41 (m, 3H), 7.28 (dd, J = 8.7, 7.3 Hz, 2H), 7.08–7.00 (m, 1H), 6.94–6.83 (m, 2H), 5.28 (t, J = 5.8 Hz, 1H), 3.12 (q, J = 6.6 Hz, 2H), 1.47–1.36 (m, 2H), 1.20–1.09 (m, 2H), and 0.79 (t, J = 7.3 Hz, 3H).

#### 4.1.10 Compounds BA-05 and BA-24

Methyl 3-(butylamino)-4-phenoxy-5-sulfamoyl-benzoate (**Int01**).

Compound **Int01** was obtained starting from bumetanide (28395–03–1) and following the procedure described in *Bioorganic Chemistry*, **2020**, 100, 103878.

3-(butylamino)-5-(hydrazinecarbonyl)-2-phenoxy-benzenesulfonamide (**Int02**).

A stirred solution of **Int01** (200 mg, 0.528 mmol, 1 equiv.) in hydrazine 1M in THF (2 mL, 2.11 mmol, 4 equiv.) was added at 70 °C for 5 days. The reaction mixture was concentrated under reduced pressure, and the crude oil was taken up in pyridine (2 mL) and stirred at 110 °C for 16 h. The reaction mixture was concentrated under reduced pressure and triturated in DCM/MeOH 9/1 to obtain **Int02** (100 mg, 0.264 mmol, 50%) as a white solid.

C17H22N4O4S; MS (ESI+) m/z: 379 [M + H]+.
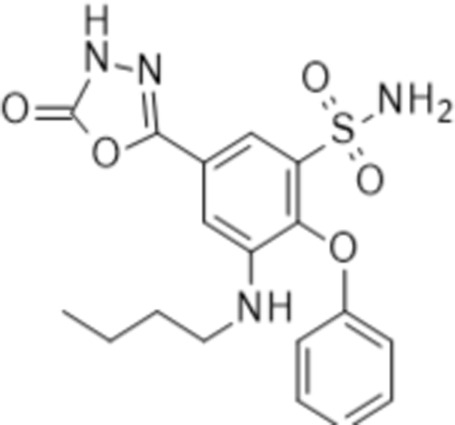


3-(butylamino)-5-(2-oxo-3H-1,3,4-oxadiazol-5-yl)-2-phenoxy-benzenesulfonamide (**compound BA-05**).

For BA-05: Triethylamine (14 µL, 0.099 mmol, 1.5 equiv.) and carbonyl diimidazole (12 mg, 0.072 mmol, 1.1 equiv.) were added to a stirred solution of **Int02** (25 mg, 0.066 mmol, 1 equiv.) in DMF (1 mL, 0.05 M), and the solution was stirred at 20 °C for 16 h. The reaction mixture was concentrated under reduced pressure, and the crude oil was purified by preparative thin-layer chromatography with DCM/MeOH 95/5 to obtain **compound BA-05** (15 mg, 0.037 mmol, 56%) as a white solid.

C18H20N4O5S; MS (ESI+) m/z: 405 [M + H]+; 1H NMR (400 MHz, DMSO-d6): δ 12.65 (s, 1H), 7.49 (d, J = 2.0 Hz, 1H), 7.40 (s, 2H), 7.34–7.23 (m, 2H), 7.20 (d, J = 2.1 Hz, 1H), 7.02 (t, J = 7.3 Hz, 1H), 6.90–6.81 (m, 2H), 5.20 (t, J = 5.8 Hz, 1H), 3.09 (q, J = 6.6 Hz, 2H), 1.43–1.32 (m, 2H), 1.20–1.08 (m, 2H), and 0.78 (t, J = 7.3 Hz, 3H).
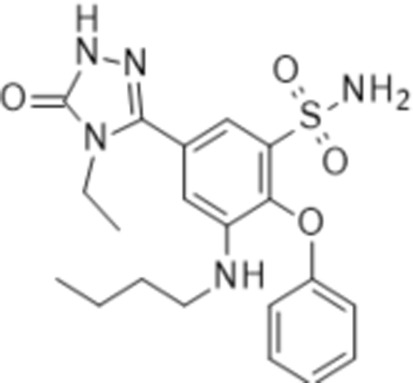


3-(butylamino)-5-(4-ethyl-5-oxo-1H-1,2,4-triazol-3-yl)-2-phenoxy-benzenesulfonamide (**compound BA-24**).

For BA-24: Ethyl isocyanate (12 µL, 0.152 mmol, 1.15 equiv.) was added to a stirred solution of **Int02** (50 mg, 0.132 mmol, 1 equiv.) in THF (2 mL, 0.05 M), and the solution was stirred at 20 °C for 1 h. An amount of 5 mL of Et_2_O was added to make precipitate a solid that was recovered by filtration and air-dried. The solid was taken up in NaOH 1M, and the orange suspension was stirred at 90 °C for 16 h. The reaction mixture was extracted twice with CHCl_3_/iPrOH 9/1, and the organic layer was dried over MgSO_4_, filtered, and concentrated to dryness to obtain **compound BA-24** (15 mg, 0.035 mmol, 26%) as a yellow solid.

C20H25N5O4S; MS (ESI+) m/z: 432 [M + H]+; 1H NMR (400 MHz, DMSO-d6): δ 11.94 (s, 1H), 7.36 (s, 1H), 7.32–7.23 (m, 3H), 7.11 (d, J = 2.0 Hz, 1H), 7.06–6.97 (m, 1H), 6.92–6.83 (m, 2H), 5.15 (t, J = 5.9 Hz, 1H), 3.75 (q, J = 7.2 Hz, 2H), 3.08 (q, J = 6.6 Hz, 2H), 1.43–1.30 (m, 2H), 1.22–1.09 (m, 6H), and 0.78 (t, J = 7.4 Hz, 3H).

#### 4.1.11 Compounds BA-27 and BA-06

3-(butylamino)-4-phenoxy-5-sulfamoyl-benzamide (**Int03**).

Carbonyl diimidazole (49 mg, 0.301 mmol, 1.1 equiv.) was aded to a stirred solution of Bumetanide (100 mg, 0.274 mmol, 1 equiv.) in acetonitrile (2.7 mL, 0.1 M), and the solution was stirred at 50 °C for 1 h; then, a 28% aqueous solution of ammoniac (42 µL, 0.302 mmol, 1.1 equiv.) was added, and the reaction mixture was stirred at 20 °C for 16 h. The reaction mixture was concentrated under reduced pressure, and the crude oil was triturated in acetonitrile to obtain **Int03** (80 mg, 0.220 mmol, 80%) as a white solid.

C17H21N3O4S; MS (ESI+) m/z: 364 [M + H]+; 1H NMR (400 MHz, DMSO-d6): δ 8.09 (s, 1H), 7.64 (d, J = 2.0 Hz, 1H), 7.42 (d, J = 2.1 Hz, 2H), 7.32–7.05 (m, 4H), 7.05–6.97 (m, 1H), 6.88–6.81 (m, 2H), 4.85 (t, J = 5.8 Hz, 1H), 3.08 (q, J = 6.6 Hz, 2H), 1.44–1.29 (m, 2H), 1.21–1.04 (m, 2H), and 0.78 (t, J = 7.3 Hz, 3H).

3-(butylamino)-5-cyano-2-phenoxy-benzenesulfonamide (**Int04**).

Dry pyridine (120 µL, 1.49 mmol, 3 equiv.) and trifluoroacetic anhydride (105 µL, 0.742 mmol, 1.5 equiv.) were added to a stirred solution of **Int03** (180 mg, 0.495 mmol, 1 equiv.) in dry dioxane (2.5 mL, 0.2 M). The solution was stirred at 20 °C for 1 h. The reaction mixture was concentrated under reduced pressure, and the crude oil was purified by automated flash chromatography with DCM/MeOH (gradient from 1/0 to 9/1 over 10 CV) to obtain **Int04** (99 mg, 0.495 mmol, 58%) as a white solid.

C17H19N3O3S; MS (ESI+) m/z: 346 [M + H]+. 1H NMR (500 MHz, DMSO-d6) δ 7.47 (s, 2H), 7.38 (t, J = 2.1 Hz, 2H), 7.33–7.23 (m, 2H), 7.07–6.98 (m, 1H), 6.88–6.80 (m, 2H), 5.42 (t, J = 5.9 Hz, 1H), 3.07 (q, J = 6.6 Hz, 2H), 1.43–1.28 (m, 2H), 1.16–1.02 (m, 2H), and 0.77 (t, J = 7.4 Hz, 3H).
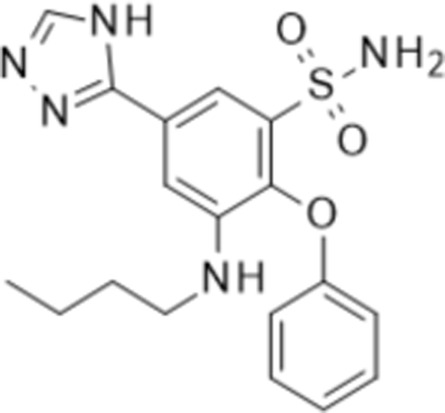


3-(butylamino)-2-phenoxy-5-(4H-1,2,4-triazol-3-yl) benzenesulfonamide (**compound BA-27**).

A stirred solution of **Int03** (150 mg, 0.413 mmol, 1 equiv.) in DMF-DMA (1 mL, 0.4 M) was stirred at 120 °C for 2 h. The reaction mixture was concentrated under reduced pressure, and the crude oil was taken up in acetic acid (1 mL, 0.4 M) and treated with hydrazine hydrate (28 µL, 0.454 mmol, 1.1 equiv.). The resulting mixture was stirred at 90 °C for 2 h. The reaction mixture was concentrated under reduced pressure, taken up in EtOAc, and poured into a saturated solution of NaHCO_3_ in water to reach a basic pH. The aqueous layer was extracted with EtOAc, and the organic layer was dried with Na_2_SO_4_, filtered, and concentrated under reduced pressure. The crude oil was purified by automated flash purification with DCM/MeOH (gradient from 1/0 to 9/1 in 4 CV). The resulting intermediate was diluted in MeOH (1 mL) and THF (2 mL) and stirred at 20 °C with solid NaOH (30 mg, 0.746 mmol) for 3 days. The reaction mixture was poured into water and extracted twice with CHCl_3_/iPrOH 8/2. The combined organic layers were dried with MgSO_4_, filtered, and concentrated under reduced pressure. The crude oil was triturated once in acetonitrile and once with methyl *tert*-butylether to obtain **compound 30** (30 mg, 0.248 mmol, 31%) as a white solid.

C18H21N5O3S; MS (ESI+) m/z: 388 [M + H]+; 1H NMR (400 MHz, DMSO-d6): δ 14.18 (s, 1H), 8.63 (s, 1H), 7.81 (d, J = 1.9 Hz, 1H), 7.56 (d, J = 1.9 Hz, 1H), 7.34–7.17 (m, 4H), 7.01 (t, J = 7.3 Hz, 1H), 6.88 (d, J = 8.1 Hz, 2H), 4.94 (s, 1H), 3.15–3.02 (m, 2H), 1.48–1.35 (m, 2H), 1.22–1.12 (m, 2H), and 0.79 (t, J = 7.3 Hz, 3H).
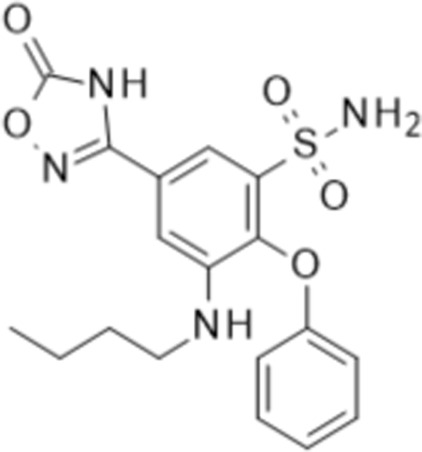


3-(butylamino)-5-(5-oxo-4H-1,2,4-oxadiazol-3-yl)-2-phenoxy-benzenesulfonamide (**compound BA-06**).

A 1.2 M aqueous solution of sodium bicarbonate (650 µL, 0.781 mmol, 3 equiv.) and hydroxylamine hydrochloride (36 mg, 0.521 mmol, 2 equiv.) were added to a stirred solution of **Int04** (90 mg, 0.260 mmol, 1 equiv.) in ethanol (2.5 mL, 0.1 M) at 20 °C. The resulting mixture was stirred at 80 °C for 4 h. The reaction mixture was poured into water; the aqueous layer was extracted with EtOAc; and the organic layer was dried with Na_2_SO_4_, filtered, and concentrated under reduced pressure. The resulting crude white solid was diluted in dioxane (2.5 mL, 0.1 M) and treated with DBU (38.5 µL, 253 mmol, 1.1 equiv.) and carbonyl diimidazole (56 mg, 0.344 mmol, 1.5 equiv.) at 20 °C. The reaction mixture was stirred at 100 °C for 16 h. The reaction mixture was concentrated under reduced pressure, and the crude oil was purified by preparative thin-layer chromatography with DCM/MeOH 95/5 to obtain **compound BA-06** (15 mg, 0.037 mmol, 16%) as a yellow solid.

C18H20N4O5S; MS (ESI+) m/z: 405 [M + H]+; 1H NMR (400 MHz, Acetonitrile-d3) δ 7.72–7.58 (m, 2H), 7.42 (s, 1H), 7.34 (t, J = 7.7 Hz, 2H), 7.06 (s, 2H), 6.94 (d, J = 8.0 Hz, 2H), 4.47–4.35 (m, 1H), 3.15–3.03 (m, 2H), 1.47–1.35 (m, 2H), 1.23–1.07 (m, 2H), and 0.81 (t, J = 7.4 Hz, 3H).

#### 4.1.12 Compounds BA-134 and BA-33

4-chloro-3-nitro-5-sulfanyl-benzoic acid acid (**Int09**).

KNO_3_ (555 mg, 5.79 mmol, 1 equiv.) was added at 0 °C to solution of **4-chloro-3-chlorosulfonyl-benzoic acid** (CAS 2494-79-3) (1.4 g, 5.49 mmol, 1 equiv.) diluted in concentrated sulfuric acid (4.4 mL, 82.33 mmol, 15 equiv.), and the resulting yellow suspension was stirred at 100 °C for 16 h. The suspension dissolved as the temperature increased. The reaction mixture was poured into 20 mL of ice/water and extracted with EtOAc; the combined organic layers were dried over Na_2_SO_4_, filtered, and concentrated under reduced pressure to obtain a nitro intermediate (793 mg, 2.64 mmol, 48%) as a white solid.

C7H3Cl2NO6S; 1H NMR (400 MHz, DMSO-d6): δ 8.63 (d, *J* = 2.1 Hz, 1H), 8.37 (d, *J* = 2.1 Hz, 1H).

Triphenylphosphine (5.25 g, 20 mmol, 3 equiv.) was added at 20 °C to a solution of the aforementioned nitro intermediate (2 g, 6.66 mmol, 1 equiv.) diluted in toluene (66.6 mL, 0.1 M), and the resulting yellow suspension was stirred at 20 °C for 1 h. Water was added, and stirring was continued for 1 h, followed by the addition of 1M NaOH and phase separation. The organic layer was washed twice with NaOH 1M. The aqueous layer was acidified to pH 3 with HCl 2M, followed by the filtration of the suspension; then, the solid was washed once with water and air dried to obtain **Int09** (400 mg, 1.54 mmol, 23%) as a white solid.

C7H4ClNO4S; MS (ESI+) m/z: 232 [M-1]-; 1H NMR (400 MHz, DMSO-d6): δ 8.55 (d, *J* = 1.7 Hz, 1H), 8.45 (s, 2H), and 8.33 (d, *J* = 1.7 Hz, 1H).

Methyl 3-methylsulfanyl-5-nitro-4-phenoxy-benzoate (**Int10**).

K_2_CO_3_ (710 mg, 5.14 mmol, 3 equiv.) and methyl iodide (234 µL, 3.77 mmol, 2.2 equiv.) were added at 20 °C to a solution of **Int09** (400 mg, 1.71 mmol, 1 equiv.) diluted in DMF (11 mL, 0.15 M), and the resulting yellow suspension was stirred at 20 °C for 16 h. The reaction mixture was poured into water and extracted with EtOAc; the combined organic layers were dried over Na_2_SO_4_, filtered, and concentrated under reduced pressure to obtain a yellow solid (220 mg, 0.80 mmol, 47%).

C9H8ClNO4S; MS (ESI+) m/z: 262, 264 [M + H]+; 1H NMR (400 MHz, DMSO-d6): δ 8.26 (d, *J* = 1.8 Hz, 1H), 7.95 (d, *J* = 1.8 Hz, 1H), 3.93 (s, 3H), and 2.67 (s, 3H).

Phenol (35.6 mg, 0.38 mmol, 1.1 equiv.) and K_2_CO_3_ (95 mg, 0.70 mmol, 2 equiv.) were added at 20 °C to a solution of the aforementioned yellow solid (90 mg, 0.34 mmol, 1 equiv.) diluted in DMF (3 mL, 0.1 M), and the resulting yellow suspension was stirred at 100 °C for 4 h. The reaction mixture was poured into water and extracted with EtOAc; the combined organic layers were dried over Na_2_SO_4_, filtered, and concentrated under reduced pressure. The crude oil was purified by automated flash chromatography with cyclohexane/EtOAc (gradient from 100/0 to 1/1 over 10 CV) to obtain **Int10** (64 mg, 0.2 mmol, 60%) as a yellow solid.

C15H13NO5S; MS (ESI+) m/z: 320 [M + H]+; 1H NMR (400 MHz, DMSO-d6): δ 8.32 (d, *J* = 2.0 Hz, 1H), 8.10 (d, *J* = 2.0 Hz, 1H), 7.38–7.31 (m, 2H), 6.91–6.87 (m, 2H), 6.80–6.71 (m, 1H), and 3.95 (s, 3H), 2.54 (s, 3H).

Methyl 3-(methylsulfonimidoyl)-5-nitro-4-phenoxy-benzoate (**Int11**).

Ammonium carbamate (264 mg, 3.38 mmol, 4 equiv.) and (diacetoxyiodo)benzene (817 mg, 2.54 mmol, 3 equiv.) were added at 20 °C to a solution of **Int10** (270 mg, 0.85 mmol, 1.00 equiv.) diluted in MeOH (8 mL, 0.1 M) and CH_2_Cl_2_ (2 mL), and the resulting yellow suspension was stirred at 20 °C for 2 h. The reaction mixture was concentrated under reduced pressure and then purified by automated flash chromatography with cyclohexane/EtOAc (gradient from 1/0 to 0/1 over 9 CV) to obtain **Int011** (276 mg, 0.75 mmol, 89%) as a yellow solid.

C15H14N2O6S; MS (ESI+) m/z: 351 [M + H]+; 1H NMR (400 MHz, DMSO-d6): δ 8.82 (d, *J* = 2.2 Hz, 1H), 8.70 (d, *J* = 2.2 Hz, 1H), 7.37–7.31 (m, 2H), 7.19–7.08 (m, 1H), 7.01–6.91 (m, 2H), 4.92 (d, *J* = 1.4 Hz, 1H), 3.97 (s, 3H), and 3.28 (d, *J* = 1.4 Hz, 3H).

Methyl 3-amino-5-(N,S-dimethylsulfonimidoyl)-4-phenoxy-benzoate (**Int12**).

**Int11** (550 mg, 1.491 mmol, 1.00 equiv.) was diluted in formic acid (5.6 mL, 149.14 mmol, 100 equiv.) and heated with paraformaldehyde (224 mg, 7.46 mmol, 5 equiv.) at 120 °C for 4 h with a microwave oven. The reaction mixture was concentrated under reduced pressure and then purified by automated flash chromatography with cyclohexane/EtOAc (gradient from 1/0 to 0/1 over 10 CV) to obtain a yellow oil (481 mg, 1.188 mmol, 80%).

C16H16N2O6S; MS (ESI+) m/z: 365 [M + H]+; 1H NMR (400 MHz, DMSO-d6) δ 8.75–8.69 (m, 2H), 7.38–7.29 (m, 2H), 7.12 (td, J = 7.3, 1.1 Hz, 1H), 7.00–6.92 (m, 2H), 3.97 (s, 3H), 3.34 (s, 3H), and 2.35 (s, 3H).

Ammonium chloride (636 mg, 11.9 mmol, 10.0 equiv.) was added to a solution of the previously obtained yellow oil (481 mg, 1.19 mmol, 1.00 equiv.) diluted in ethanol (8.3 mL, 0.1 M) and water (4.2 mL, 0. 1 M). The suspension was stirred at 85 °C for 10 min. Then, iron (0.93 g, 16.6 mmol, 14.0 equiv.) was added at once. The reaction was stirred at 85 °C for 2 h. The mixture was cooled down to 20 °C. Water was added, and the organic layer was extracted with EtOAc (thrice). The combined organic layers were dried over Na_2_SO_4_, filtered, and concentrated under reduced pressure to obtain **Int12** (404 mg, 1.01 mmol, 85%) as a brown oil.

C16H18N2O4S; MS (ESI+) m/z: 335 [M+1]+; 1H NMR (400 MHz, DMSO-d6): δ 7.78–7.62 (m, 2H), 7.29 (t, J = 7.8 Hz, 2H), 7.03 (t, J = 7.4 Hz, 1H), 6.80 (d, J = 8.0 Hz, 2H), 5.46 (s, 2H), 3.87 (s, 3H), 3.16 (s, 3H), and 2.19 (s, 3H).
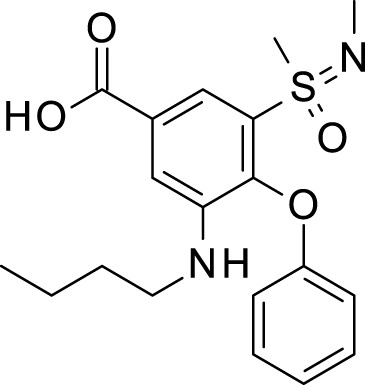


3-(butylamino)-5-(N,S-dimethylsulfonimidoyl)-4-phenoxy-benzoic acid (**compound BA-134**).

Butyraldehyde (207 µL, 2.29 mmol, 3.00 equiv.) and then sodium triacetoxyborohydride (324 mg, 1.53 mmol, 2.00 equiv.) were added at 20 °C to a solution of **Int12** (278 mg, 0.765 mmol, 1.00 equiv.) diluted in 1,2-dichloroethane (5.1 mL, 0.15 M). The resulting solution was stirred at 20 °C for 7 h. After the addition of water, the aqueous layer was extracted thrice with DCM. The combined organic layers were dried over Na_2_SO_4_, filtered, and concentrated under reduced pressure to obtain a crude compound. The crude compound was purified by automated flash chromatography with cyclohexane/EtOAc (gradient from 1/0 to 0/1 over 10 CV) to obtain a yellow oil (203 mg, 0.468 mmol, 61%).

C20H26N2O4S; MS (ESI+) m/z: 391 [M+1]+; 1H NMR (400 MHz, DMSO-d6): δ 7.70 (d, J = 2.0 Hz, 1H), 7.48 (d, J = 2.1 Hz, 1H), 7.32–7.26 (m, 2H), 7.07–7.00 (m, 1H), 6.82–6.77 (m, 2H), 5.31 (t, J = 5.7 Hz, 1H), 3.89 (s, 3H), 3.16 (s, 3H), 3.09 (q, J = 6.6 Hz, 2H), 2.17 (s, 3H), 1.46–1.34 (m, 2H), 1.16 (dt, J = 12.1, 7.2 Hz, 2H), and 0.80 (t, J = 7.3 Hz, 3H).

According to **GP2** (method A), starting from the previously isolated yellow oil, **compound BA-134** was isolated as a yellow solid (258 mg, 0.6444 mmol, 99%).

C19H24N2O4S; MS (ESI+) m/z: 377 [M + H]+; 1H NMR (400 MHz, DMSO-d6) δ 7.78–7.70 (m, 1H), 7.55 (s, 1H), 7.33–7.24 (m, 2H), 7.02 (t, J = 7.3 Hz, 1H), 6.79 (d, J = 8.0 Hz, 2H), 4.96–4.82 (m, 1H), 3.13 (s, 3H), 3.11–3.04 (m, 2H), 2.19 (s, 3H), 1.46–1.36 (m, 2H), 1.20–1.12 (m, 2H), and 0.80 (t, J = 7.3 Hz, 3H).

13C NMR (101 MHz, DMSO) δ 162.3, 156.6 (2C), 142.6, 132.5, 129.7 (2C), 122.6, 118.7, 118.5, 116.6, 115.5 (2C), 43.6, 42.7, 30.9, 29.7, 19.9, and 14.1.
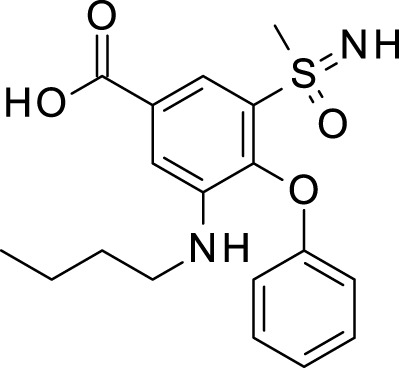


3-(butylamino)-5-(methylsulfonimidoyl)-4-phenoxy-benzoic acid (**compound BA-33**).

Palladium 10% sur charcoal (80 mg, 0.07 mmol, 0.100 equiv.) was added to a stirred solution of **Int11** (276 mg, 0.75 mmol, 1.00 equiv.) in MeOH (7.5 mL, 0.1 M) under argon, and the resulting black suspension was stirred under 1 atm H_2_ at 20 °C for 16 h. The reaction mixture was filtered over celite, and the filtrate was concentrated under reduced pressure. The crude oil was taken up in 1,2-dichloroethane (7.5 mL, 0.1 M) and treated with butyraldehyde (276 µL, 2.99 mmol, 4.00 equiv.) and sodium triacetoxyborohydride (280 mg, 1.49 mmol, 2.00 equiv.), and the resulting yellow suspension was stirred at 20 °C for 4 h, followed by the addition of water to the reaction mixture and extraction with CH_2_Cl_2_. The organic layer was dried over Na_2_SO_4_, filtered, and concentrated under reduced pressure. The crude oil was purified by preparative TLC with CH_2_Cl_2_/MeOH 95/5 to obtain a yellow oil.

C19H24N2O4S; MS (ESI+) m/z: 377 [M + H]+; 1H NMR (400 MHz, Chloroform-d) δ 7.93 (d, *J* = 2.0 Hz, 1H), 7.52 (d, *J* = 2.0 Hz, 1H), 7.29–7.18 (m, 2H), 7.00 (t, *J* = 7.4 Hz, 1H), 6.79 (d, *J* = 8.1 Hz, 2H), 3.87 (s, 3H), 3.13 (s, 3H), 3.8–3.01 (m, 2H), 1.44–1.31 (m, 2H), 1.17–1.03 (m, 2H), and 0.76 (t, *J* = 7.3 Hz, 3H).

A 2M aqueous solution of sodium hydroxide (104 µL, 0.18 mmol, 6 equiv.) was added at 20 °C to a stirred solution of the previously isolated yellow oil (13 mg, 0.03 mmol, 1 equiv.) in THF (690 µL, 0.05 M), and the resulting solution was stirred at 20 °C for 16 h. THF was removed, and the mixture was diluted in water (5 mL). The aqueous layer was washed with EtOAc and acidified with HCl 1N until pH 2–3. The aqueous layer was extracted with EtOAc, and the resulting organic layer was dried over Na_2_SO_4_, filtered, and concentrated under reduced pressure to obtain **compound BA-33** (2 mg, 0.005 mmol, 14%) as a yellow oil.

C18H22N2O4S; MS (+) m/z: 363 [M+H]+, MS (-) m/z: 361 [M-H]-; 1H NMR (400 MHz, CD3CN) δ 7.87 (d, *J* = 2.0 Hz, 1H), 7.58 (d, *J* = 2.0 Hz, 1H), 7.39–7.28 (m, 2H), 7.14–7.06 (m, 1H), 6.93–6.86 (m, 2H), 4.37 (brs, 1H), 3.19 (s, 3H), 3.14 (t, *J* = 7.0 Hz, 2H), 1.99 (s, 1H), 1.49–1.38 (m, 2H), 1.24–1.12 (m, 2H), and 0.83 (t, *J* = 7.3 Hz, 3H).

#### 4.1.13 Compound I-03

Methyl 4-fluoro-3-(methylsulfonimidoyl)benzoate (**Int13**).

Ammonium carbamate (195 mg, 2.50 mmol, 3 equiv.) and PIDA (600 mg, 1.87 mmol, 4 equiv.) were added to a stirred solution of **methyl 4-fluoro-3-(methylthio)benzoate** (125 mg, 0.62 mmol, 1 equiv.) in MeOH. The mixture was stirred for 2 h at 20 °C. The solvent was removed under reduced pressure, and the crude residue was purified by automated flash chromatography with cyclohexane/EtOAc (gradient from 95/5 to 0/100 over 12 CV) to obtain **Int13** as a colorless oil (129 mg, 0.552 mmol, 88%).

C9H10FNO3S; MS (ESI+) m/z: 232 [M + H]+; 1H NMR (400 MHz, Chloroform-d) δ 8.63 (dd, J = 7.0 Hz, J = 2.2 Hz, 1H), 8.29 (ddd, J = 8.6 Hz, J = 4.7 Hz, J = 2.3 Hz, 1H), 7.31 (dd, J = 9.3 Hz, J = 8.6 Hz, 1H), 3.95 (s, 3H), 3.29 (s, 3H), and 3.09 (br s, 1H). 19F NMR (400 MHz, chloroform-d): δ −102.3.

Methyl 3-(methylsulfonimidoyl)-4-(8,8,8-trifluorooctylamino)benzoate (**Int14**).

Hydrazine monohydrate (277 µL, 5.58 mmol, 10 equiv.) was added to a stirred solution of **2-(8,8,8-trifluorooctyl)isoindoline-1,3-dione** (prepared following (Savardi et al., 2020) 262 mg, 0.83 mmol, 1.5 equiv.) in dry EtOH (10 mL). The solution was stirred for 1 h at 80 °C, and the mixture was cooled down to 20 °C. The suspension was filtered, the solid was washed with DCM, and the filtrate was concentrated under reduced pressure. The residue was taken up in dioxane (5 mL) and was added to **Int13** (129 mg, 0.56 mmol). The solution was stirred for 16 h at 110 °C. After cooling down, the mixture was partitioned between an aqueous saturated solution of ammonium chloride and EtOAc. The aqueous layer was extracted with EtOAc twice, and the combined organic layers were washed with brine, filtered over sodium sulfate, and concentrated under reduced pressure. The crude oil was purified by automated flash chromatography with cyclohexane/EtOAc (gradient from 100/0 to 30/70 over 10 CV) to obtain **Int14** as a sticky solid (145 mg, 0.37 mmol, 66%).

C17H25F3N2O3S; MS (ESI+) m/z: 395 [M + H]+; 1H NMR (400 MHz, chloroform-d) δ 8.50 (d, J = 2.1 Hz, 1H), 8.05 (dd, J = 8.8 Hz, J = 2.1 Hz, 1H), 7.46 (t, J = 4.8 Hz, 1H), 6.71 (d, J = 8.8 Hz, 1H), 3.87 (s, 3H), 3.26–3.18 (m, 2H), 3.08 (d, J = 1.2 Hz, 3H), 2.85 (s, 1H), 2.13–1.99 (m, 2H), 1.75–1.66 (m, 2H), 1.61–1.51 (m, 2H), and 1.48–1.35 (m, 6H). 19F NMR (376 MHz, chloroform-d) δ −66.4.
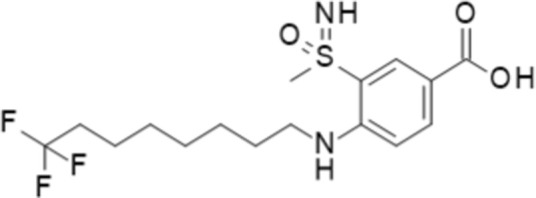


3-(methylsulfonimidoyl)-4-(8,8,8-trifluorooctylamino)benzoic acid (**compound I-03**).

LiOH (20 mg, 0.72 mmol, 2 equiv.) was added to a solution of **Int14** (145 mg, 0.36 mmol, 1 equiv.) in a mixture of THF/H_2_O/MeOH 1/1/1 (6 mL). The resulting mixture was stirred for 16 h at 20 °C. THF was removed under reduced pressure, and the mixture was diluted in water (5 mL). The aqueous layer was washed with EtOAc and acidified with a 1 M aqueous solution of HCl until pH 2–3. The aqueous layer was extracted with EtOAc, and the resulting organic layer was dried over sodium sulfate, filtered, and concentrated under reduced pressure to obtain **compound I-03** (94 mg, 0.25 mmol, 68%) as a white solid.

C16H23F3N2O3S; MS (ESI+) m/z: 381 [M + H]+; 1H NMR (400 MHz, DMSO-d6): δ 8.26 (d, J = 2.1 Hz, 1H), 7.92 (dd, J = 8.7 Hz, J = 2.1 Hz, 1H), 7.63 (t, J = 5.3 Hz, 1H), 6.85 (d, J = 8.8 Hz, 1H), 4.63 (s, 1H), 3.31 (s, 1H), 3.27–3.18 (m, 2H), 3.02 (s, 3H), 2.33–2.14 (m, 2H), 1.67–1.55 (m, 2H), 1.53–1.42 (m, 2H), and 1.41–1.28 (m, 6H). 19F NMR (376 MHz, DMSO-d6): δ −64.7.

13C NMR (101 MHz, DMSO-d6): δ 166.6, 149.6, 135.3, 131.9, 129.1, 122.5, 111.3, 43.8, 42.2, 32.5 (q, J = 27 Hz), 28.2, 28.1, 27.8, 26.2, and 21.3 (q, J = 3 Hz). (CF3 is missing: signal in the background.)

#### 4.1.14 Compounds from table 3

Methyl 3-(butylamino)-4-phenoxy-5-sulfamoyl-benzoate (**Int06**).

Compound **Int06** was obtained starting from bumetanide (28395-03-1) following the procedure described in Ibrahim et al. (2020).

Methyl 3-(butylamino)-5-[[tert-butyl(dimethyl)silyl]sulfamoyl]-4-phenoxy-benzoate (**Int07**).

Ttriethylamine (940 µL, 6.75 mmol, 2.2 equiv.) was added to a stirred suspension of **Int06** (1.16 g, 3.06 mmol) in dry THF (4 mL, 0.8 M) under an argon atmosphere. The mixture was stirred for 10 min at 20 °C, and a solution of TBDMSCl (610 mg, 3.83 mmol, 1.25 equiv.) in toluene (1 mL) was added dropwise. The resulting mixture was stirred at 50 °C for 18 h. The solution was cooled down to 20 °C, and the resulting suspension was filtered. The solid was washed with Et_2_O, and the combined filtrates were concentrated under reduced pressure. The resulting crude residue was stirred for 5 min in a mixture of THF/Et_2_O (2/1, 12 mL), and the suspension was filtered. The filtrate was concentrated under reduced pressure to obtain **Int07** (1.50 g, 2.90 mmol, 94%) as an orange oil.

C24H36N2O5SiS; MS (ESI+) m/z: 493 [M + H]+; 1H NMR (400 MHz, Chloroform-d): δ 7.95 (d, *J* = 2.0 Hz, 1H), 7.54 (d, *J* = 2.0 Hz, 1H), 7.30 (dd, *J* = 8.7 Hz, *J* = 7.4 Hz, 2H), 7.14–7.04 (m, 1H), 6.93–6.89 (m, 2H), 4.45 (brs, 1H), 3.94 (s, 3H), 3.83 (t, *J* = 5.4 Hz, 1H), 3.11–3.09 (m, 2H), 1.45–1.38 (m, 2H), 1.20–1.10 (m, 2H), 0.85 (s, 9H), 0.81 (t, *J* = 7.3 Hz, 3H), and 0.12 (s, 6H).
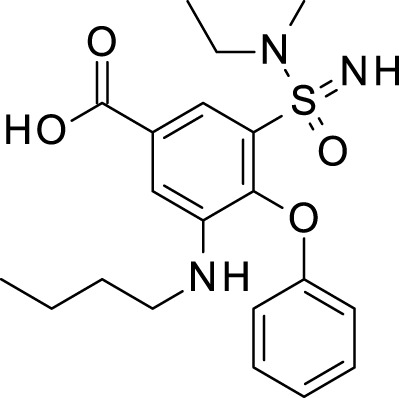


3-(butylamino)-5-[[ethyl(methyl)amino]sulfonimidoyl]-4-phenoxy-benzoic acid (**compound BA-66**).

According to **GP1** (method A), starting from **Int02** and N-methylethanamine, a white solid was isolated, namely, **BA-66-E** (102.4 mg, 0.1346 mmol, 25%).

C21H29N3O4S; MS (ESI+) m/z: 420 [M + H]+; 1H NMR (400 MHz, Chloroform-d): δ 8.01 (d, *J* = 2.0 Hz, 1H), 7.52 (d, *J* = 2.0 Hz, 1H), 7.33–7.26 (m, 2H), 7.09–7.02 (m, 1H), 6.86–6.79 (m, 2H), 3.93 (s, 3H), 3.86 (t, *J* = 5.5 Hz, 1H), 3.28–3.05 (m, 4H), 2.77 (s, 3H), 2.73 (s, 1H), 1.42 (m, 2H), 1.22–1.11 (m, 2H), 1.07 (t, *J* = 7.1 Hz, 3H), and 0.82 (t, *J* = 7.3 Hz, 3H).

According to **GP2** (method A), starting from the isolated compound, **compound BA-66** was isolated as a white solid (24 mg, 0.0586 mmol, 99%).

C20H27N3O4S; MS (ESI+) m/z: 406 [M + H]+; 1H NMR (400 MHz, DMSO-d6): δ 13.12 (s, 1H), 7.81 (d, *J* = 2.0 Hz, 1H), 7.40 (d, *J* = 2.0 Hz, 1H), 7.31–7.23 (m, 2H), 7.05–6.98 (m, 1H), 6.81–6.75 (m, 2H), 4.89 (t, *J* = 5.7 Hz, 1H), 4.08 (s, 1H), 3.16–2.91 (m, 4H), 2.65 (s, 3H), 1.41–1.30 (m, 2H), 1.17–1.04 (m, 2H), 0.98 (t, *J* = 7.1 Hz, 3H), and 0.76 (t, *J* = 7.3 Hz, 3H).

13C NMR (101 MHz, DMSO-d6): δ 166.7 156.0, 142.5, 139.5, 135.2, 129.2 (2C), 127.9, 122.1, 117.9, 115.2 (2C), 114.5, 44.4, 42.1, 34.1, 30.1, 19.3, 13.6, and 13.3.
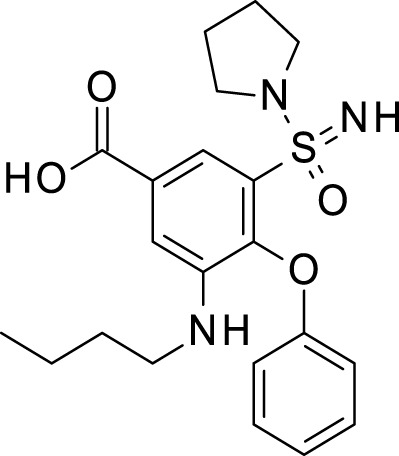


3-(butylamino)-4-phenoxy-5-(pyrrolidin-1-ylsulfonimidoyl)benzoic acid (**compound BA-51**).

According to **GP1** (method A), starting from **Int02** and pyrrolidine, a white solid was isolated (25 mg, 0.06 mmol, 7.5%).

C22H29N3O4S; MS (ESI+) m/z: 432 [M + H]+.

According to **GP2** (method A), starting from the isolated compound, **compound BA-51** was isolated as a white solid (22 mg, 0.055 mmol, 89%).

C21H27N3O4S; MS (ESI+) m/z: 418 [M + H]+; 1H NMR (400 MHz, CD3OD): δ 7.93 (d, *J* = 2.0 Hz, 1H), 7.59 (d, *J* = 2.0 Hz, 1H), 7.37–7.26 (m, 2H), 7.09–7.04 (m, 1H), 6.93–6.82 (m, 2H), 3.25–3.17 (m, 4H), 3.13 (t, *J* = 6.9 Hz, 2H), 1.72–1.58 (m, 4H), 1.50–1.38 (m, 2H), 1.24–1.12 (m, 2H), and 0.83 (t, *J* = 7.4 Hz, 3H).

13C NMR (101 MHz, CD3OD): δ 168.8, 157.5, 144.4, 141.3, 136.2, 130.8 (2C), 129.7, 124.0, 119.2, 117.1, 116.2 (2C), 48.7 (2C), 43.7, 32.0, 26.5 (2C), 20.9, and 14.
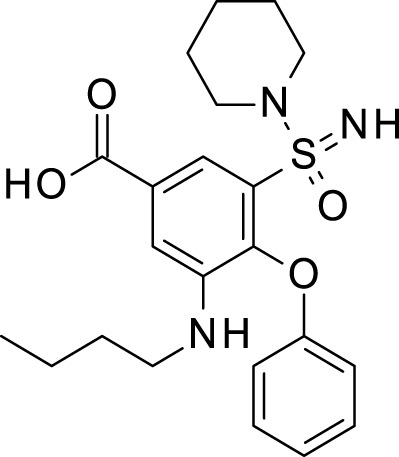


3-(butylamino)-4-phenoxy-5-(1-piperidylsulfonimidoyl)benzoic acid (**compound BA-46**).

According to **GP1** (method A), starting from **Int02** and piperidine, a sticky white solid was isolated (150 mg, 0.34 mmol, 35%).

C23H31N3O4S; MS (ESI+) m/z: 446 [M + H]+; 1H NMR (400 MHz, chloroform-d): δ 8.01 (d, *J* = 2.0 Hz, 1H), 7.52 (d, *J* = 2.0 Hz, 1H), 7.31–7.26 (m, 2H), 7.08–7.01 (m, 1H), 6.84–6.79 (m, 2H), 3.93 (s, 3H), 3.89 (t, *J* = 5.5 Hz, 1H), 3.21–3.04 (m, 6H), 2.71 (brs, 1H), 1.53–1.46 (m, 4H), 1.46–1.38 (m, 4H), 1.23–1.12 (m, 2H), and 0.82 (t, *J* = 7.3 Hz, 3H).

According to **GP2** (method A), starting from the isolated compound, **compound BA-46** was isolated as a white solid (105 mg, 0.24 mmol, 90%).

C22H29N3O4S; MS (ESI+) m/z: 432 [M + H]+; 1H NMR (400 MHz, DMSO-d6): δ 13.12 (s, 1H), 7.78 (d, *J* = 2.0 Hz, 1H), 7.40 (d, *J* = 2.0 Hz, 1H), 7.31–7.23 (m, 2H), 7.03–6.97 (m, 1H), 6.79–6.72 (m, 2H), 4.97 (t, *J* = 5.7 Hz, 1H), 4.06 (brs, 1H), 3.09–3.02 (m, 2H), 3.01–2.92 (m, 4H), 1.48–1.30 (m, 8H), 1.15–1.05 (m, 2H), and 0.77 (t, *J* = 7.3 Hz, 3H).

13C NMR (101 MHz, DMSO-d_6_): δ 167.6, 157.1, 143.6, 140.7, 134.6, 130.1 (2C), 128.9, 122.9, 119.1, 116.1 (2C), 115.5, 47.5 (2C), 43.0, 31.1, 26.2 (2C), 24.3, 20.2, and 14.5.
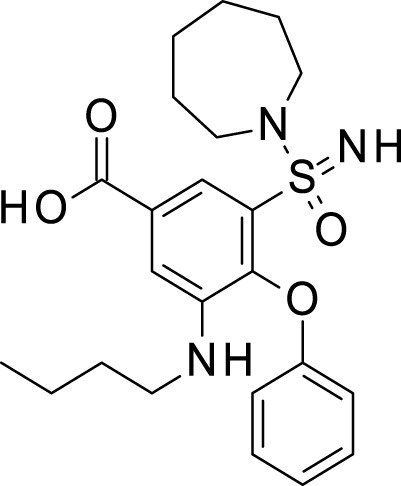


3-(azepan-1-ylsulfonimidoyl)-5-(butylamino)-4-phenoxy-benzoic acid (**compound BA-70**).

According to **GP1** (method A), starting from **Int02** and azepane (0.23 mL, 2.02 mmol), **BA-70-E** was isolated as a white solid (50 mg, 0.1012 mmol, 15%).

C24H33N3O4S; MS (ESI+) m/z: 460 [M + H]+; 1H NMR (400 MHz, Chloroform-d): δ 7.98 (d, *J* = 2.0 Hz, 1H), 7.49 (d, *J* = 2.0 Hz, 1H), 7.31–7.25 (m, 2H), 7.08–7.02 (m, 1H), 6.86–6.81 (m, 2H), 3.92 (s, 3H), 3.85–3.78 (m, 1H), 3.36–3.26 (m, 2H), 3.26–3.17 (m, 2H), 3.12–3.04 (m, 2H), 1.74–1.60 (m, 3H), 1.60–1.53 (m, 4H), 1.45–1.34 (m, 2H), 1.28–1.21 (m, 1H), 1.20–1.07 (m, 2H), and 0.80 (t, *J* = 7.3 Hz, 3H).

According to **GP2** (method A), starting from the isolated compound, **compound BA-70** was isolated as a white solid (37 mg, 0.077 mmol, 71%).

C23H31N3O4S; MS (ESI+) m/z: 446 [M + H]+; 1H NMR (400 MHz, DMSO-d6): δ 13.09 (s, 1H), 7.81 (d, *J* = 2.0 Hz, 1H), 7.38 (d, *J* = 2.0 Hz, 1H), 7.32–7.25 (m, 2H), 7.08–6.98 (m, 1H), 6.81–6.76 (m, 2H), 4.88–4.84 (m, 1H), 4.04 (s, 1H), 3.24–3.15 (m, 2H), 3.24–3.06 (m, 2H), 3.06–2.99 (m, 2H), 1.59–1.45 (m, 8H), 1.38–1.29 (m, 2H), 1.16–1.03 (m, 2H), and 0.76 (t, *J* = 7.4 Hz, 3H).

13C NMR (101 MHz, DMSO-d6): δ 166.7, 155.9, 142.3, 139.4, 136.1, 129.3 (2C), 127.7, 122.2, 117.7, 115.2 (2C), 114.4, 48.3 (2C), 42.1, 30.1, 29.2 (2C), 26.3 (2C), 19.2, and 13.5.
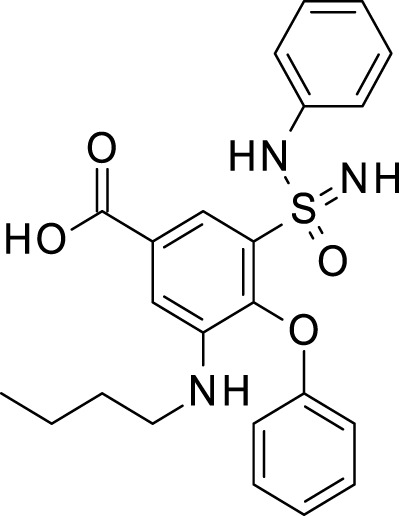


3-(anilinosulfonimidoyl)-5-(butylamino)-4-phenoxy-benzoic acid (**compound BA-64**).

According to **GP1** (method A), starting from **Int02** and aniline, a white solid was isolated (86 mg 0.1877 mmol, 19%).

C24H27N3O4S; MS (ESI+) m/z: 454 [M + H]+; 1H NMR (400 MHz, Chloroform-d): δ 7.98 (d, *J* = 2.0 Hz, 1H), 7.54 (d, *J* = 2.0 Hz, 1H), 7.31–7.24 (m, 2H), 7.05 (td, *J* = 7.4, 1.1 Hz, 1H), 6.85–6.75 (m, 2H), 3.92 (s, 4H), 3.61–3.48 (m, 4H), 3.21–3.07 (m, 6H), 1.50–1.37 (m, 3H), 1.25–1.09 (m, 2H), and 0.82 (t, *J* = 7.3 Hz, 3H).

According to **GP2** (method A), starting from the isolated intermediate, **compound BA-64** was isolated as a white solid (46 mg, 0.100 mmol, 91%).

C23H25N3O4S; MS (ESI+) m/z: 440 [M + H]+; 1H NMR (400 MHz, DMSO-d6): δ 12.95 (s, 1H), 7.86 (d, *J* = 2.0 Hz, 1H), 7.42 (d, *J* = 2.1 Hz, 1H), 7.29–7.20 (m, 2H), 7.06–6.96 (m, 3H), 6.84 (d, *J* = 8.0 Hz, 2H), 6.79–6.71 (m, 1H), 6.57 (d, *J* = 7.9 Hz, 2H), 5.07–5.00 (m, 1H), 3.12–3.04 (m, 2H), 1.43–1.32 (m, 2H), 1.19–1.05 (m, 2H), and 0.78 (t, *J* = 7.3 Hz, 3H).

13C NMR (101 MHz, DMSO-d6): δ 166.6, 156, 142.5, 139.6, 137.8, 128.9 (2C), 128.9 (3C), 127.8, 122.5, 122, 120.4 (2C), 116, 115.7 (2C), 114.5, 42, 30.1, 19.3, and 13.6.
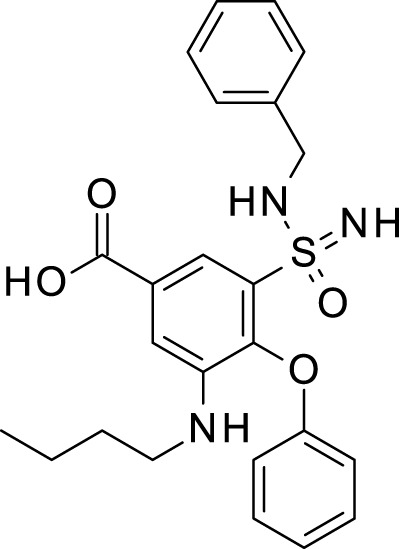


3-[(benzylamino)sulfonimidoyl]-5-(butylamino)-4-phenoxy-benzoic acid (**compound BA-63**).

According to **GP1** (method A), starting from **Int02** and benzylamine, a white solid was isolated (178 mg, 0.3699 mmol, 38%).

C25H29N3O4S; MS (ESI+) m/z: 468 [M + H]+; 1H NMR (400 MHz, chloroform-d): δ 8.07 (d, *J* = 2.0 Hz, 1H), 7.55 (d, *J* = 2.0 Hz, 1H), 7.31–7.19 (m, 5H), 7.17–7.11 (m, 2H), 7.08–7.02 (m, 1H), 6.90–6.81 (m, 2H), 4.18–4.10 (m, 1H), 3.95 (s, 3H), 3.98–3.91 (m, 1H), 3.88–3.82 (m, 1H), 3.114–3.08 (m, 2H), 1.49–1.38 (m, 2H), 1.27–1.11 (m, 2H), and 0.84 (t, *J* = 7.3 Hz, 3H).

According to **GP2** (method A), starting from the isolated intermediate, **compound BA-63** was isolated as a white solid (43 mg, 0.092 mmol, 86%).

C24H27N3O4S; MS (ESI+) m/z: 454 [M + H]+; 1H NMR (400 MHz, DMSO-d6): δ 7.80 (d, *J* = 2.0 Hz, 1H), 7.37 (d, *J* = 2.0 Hz, 1H), 7.30–7.14 (m, 7H) 7.04–6.98 (m, 1H), 6.89–6.82 (m, 2H), 4.88 (t, *J* = 5.7 Hz, 1H), 4.03 (s, 2H), 3.05 (q, *J* = 6.6 Hz, 2H), 1.43–1.30 (m, 2H), 1.18–1.06 (m, 2H), and 0.79 (t, *J* = 7.3 Hz, 3H).

13C NMR (101 MHz, DMSO-d6): δ 166.7, 156.1, 142.3, 139.6, 137.4, 129.1 (2C), 127.9 (3C), 127.4 (3C), 126.6, 122.1, 116.6, 115.5 (2C), 114.3, 46.7, 42.1, 30.2, 19.3, and 13.6.
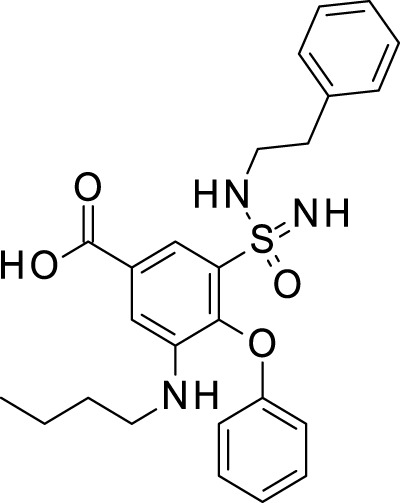


3-(butylamino)-4-phenoxy-5-[(2-phenylethylamino)sulfonimidoyl]benzoic acid (**compound BA-130**).

According to **GP1** (method B), starting from **Int02** and 2-phenylethanamine, an off-white solid was isolated (73 mg, 0.1258 mmol, 83%).

C26H31N3O4S; MS (ESI+) m/z: 482 [M + H]+; 1H NMR (400 MHz, Chloroform-d): δ 8.02 (d, *J* = 2.0 Hz, 1H), 7.55 (d, *J* = 2.0 Hz, 1H), 7.30–7.26 (m, 3H), 7.26–7.21 (m, 2H), 7.10–7.06 (m, 3H), 6.80–6.75 (m, 2H), 3.94 (s, 3H), 3.82 (s, 1H), 3.27–3.19 (m, 1H), 3.11 (t, *J* = 7.0 Hz, 2H), 3.08–2.99 (m, 1H), 2.74–2.58 (m, 2H), 1.48–1.38 (m, 2H), 1.24–1.11 (m, 2H), and 0.83 (t, *J* = 7.3 Hz, 3H).

According to **GP2** (method A), starting from the isolated compound, **compound BA-130** was isolated as a white solid (25 mg, 0.0543 mmol, 97%).

C25H29N3O4S; MS (ESI+) m/z: 468 [M + H]+; 1H NMR (400 MHz, DMSO-d6) δ 7.78 (d, *J* = 2.0 Hz, 1H), 7.38 (d, *J* = 2.0 Hz, 1H), 7.31–7.22 (m, 4H), 7.21–7.15 (m, 1H), 7.14–7.08 (m, 2H), 7.01 (t, *J* = 7.3 Hz, 1H), 6.84–6.79 (m, 2H), 4.91 (t, *J* = 5.8 Hz, 1H), 3.09–2.93 (m, 4H), 2.58 (t, *J* = 7.7 Hz, 2H), 1.41–1.31 (m, 2H), 1.17–1.04 (m, 2H), and 0.76 (t, *J* = 7.3 Hz, 3H).

13C NMR (101 MHz, DMSO-d6): δ 167.15, 156.60, 142.83, 140.14, 137.48, 129.65 (2C), 129.01 (3C), 128.77 (2C), 128.39, 126.56, 122.68, 117.20, 115.94 (2C), 114.82, 45.19, 42.55, 36.31, 30.66, 19.77, and 14.07.
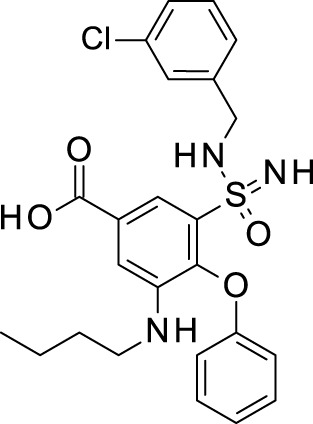


3-(butylamino)-5-[[(3-chlorophenyl)methylamino]sulfonimidoyl]-4-phenoxy-benzoic acid (**compound BA-101**).

According to **GP1** (method B), starting from **Int02** and (3-chlorophenyl)methanamine, a white solid was isolated (262 mg, 0.5219 mmol, 99%).

C25H28ClN3O4S; MS (ESI+) m/z: 502 [M + H]+; 1H NMR (400 MHz, chloroform-d): δ 8.01 (d, *J* = 2.0 Hz, 1H), 7.55 (d, *J* = 1.9 Hz, 1H), 7.32–7.26 (m, 2H), 7.18–7.12 (m, 3H), 7.10–7.03 (m, 2H), 6.92–6.86 (m, 2H), 4.19–3.96 (m, 2H), 3.95 (s, 3H), 3.10 (t, *J* = 7.0 Hz, 2H), 1.50–1.38 (m, 2H), 1.23–1.14 (m, 2H), and 0.84 (t, *J* = 7.3 Hz, 3H).

According to GP2 (method A), starting from the isolated intermediate, compound BA-101 was isolated as a white solid (238 mg, 0.4828 mmol, 93%).

C24H26ClN3O4S; MS (ESI+) m/z: 488 [M + H]+; 1H NMR (400 MHz, DMSO-d6): δ 7.78 (d, *J* = 2.0 Hz, 1H), 7.35 (d, *J* = 2.0 Hz, 1H), 7.29–7.18 (m, 5H), 7.15 (d, *J* = 7.1 Hz, 1H), 7.00 (t, *J* = 7.3 Hz, 1H), 6.87–6.83 (m, 2H), 4.82 (t, *J* = 5.4 Hz, 1H), 4.04 (s, 2H), 3.03 (q, *J* = 6.4 Hz, 2H), 1.41–1.32 (m, 2H), 1.17–1.06 (m, 2H), and 0.78 (t, *J* = 7.3 Hz, 3H).

13C NMR (101 MHz, DMSO-d6): δ 167, 156, 142, 140, 137, 133 (2C), 130, 129 (2C), 128, 127, 126, 123, 117, 116 (2C), 115, 46.4, 42.4, 30.6, 19.6, and 14.0.
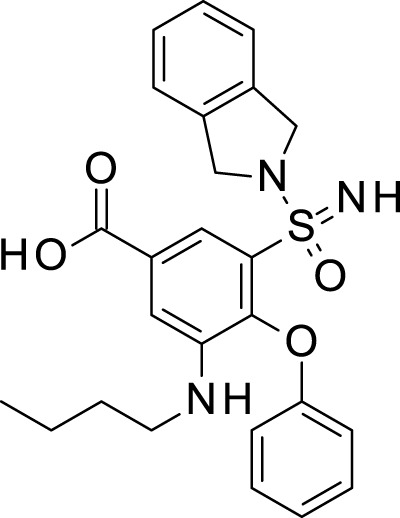


3-(butylamino)-5-(isoindolin-2-ylsulfonimidoyl)-4-phenoxy-benzoic acid (**compound BA-102**).

According to **GP1** (method B), starting from **Int02** and isoindolin, a brown oil was isolated (239 mg, 0.4834 mmol, 97%).

C26H29N3O4S; MS (ESI+) m/z: 480 [M + H]+; 1H NMR (400 MHz, Chloroform-d): δ 8.04 (d, *J* = 2.0 Hz, 1H), 7.51 (d, *J* = 2.0 Hz, 1H), 7.24–7.16 (m, 4H), 7.14–7.09 (m, 2H), 6.99–6.94 (m, 1H), 6.85–6.80 (m, 2H), 4.71–4.61 (m, 4H), 3.91 (s, 3H), 3.07 (t, *J* = 7.0 Hz, 2H), 1.44–1.35 (m, 2H), 1.20–1.09 (m, 2H), and 0.80 (t, *J* = 7.4 Hz, 3H).

According to **GP2** (method A), starting from the isolated compound, **compound BA-102** was isolated as a brown foam (242 mg, 0.5042 mmol, 95%).

C25H27N3O4S; MS (ESI+) m/z: 466 [M + H]+; 1H NMR (400 MHz, DMSO-d6): δ 13.09 (s, 1H), 7.89 (d, *J* = 2.0 Hz, 1H), 7.38 (d, *J* = 2.0 Hz, 1H), 7.22 (s, 4H), 7.21–7.18 (m, 2H), 6.93 (t, *J* = 7.4 Hz, 1H), 6.80–6.77 (m, 2H), 4.89 (t, *J* = 5.7 Hz, 1H), 4.60–4.47 (m, 4H), 2.99 (q, *J* = 6.4 Hz, 2H), 1.35–1.27 (m, 2H), 1.10–1.00 (m, 2H), and 0.72 (t, *J* = 7.3 Hz, 3H).

13C NMR (101 MHz, DMSO-d6): δ 167, 156, 142, 140, 137, 133 (2C), 130, 129 (2C), 128, 127, 126, 123, 117, 116 (2C), 115, 46.4, 42.4, 30.6, 19.6, and 14.0.
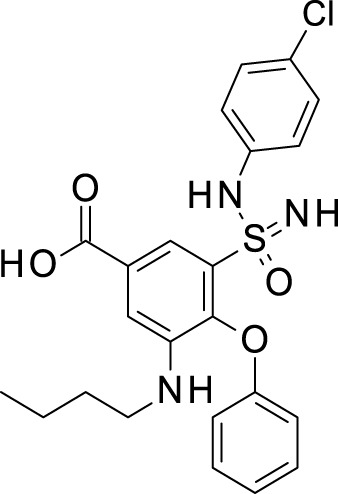


3-(butylamino)-5-[(4-chloroanilino)sulfonimidoyl]-4-phenoxy-benzoic acid (**compound BA-115**).

According to **GP1** (method B), starting from **Int02** and 4-chloroaniline, a white solid was isolated (97 mg, 0.1990 mmol, 43%).

C24H26ClN3O4S; MS (ESI+) m/z: 488 [M + H]+; 1H NMR (400 MHz, chloroform-d): δ 8.09 (d, *J* = 2.0 Hz, 1H), 7.58 (d, *J* = 2.0 Hz, 1H), 7.35–7.27 (m, 2H), 7.12 (t, *J* = 7.4 Hz, 1H), 7.07–7.00 (m, 2H), 6.93–6.85 (m, 2H), 6.71–6.58 (m, 2H), 3.95 (s, 3H), 3.12 (t, *J* = 7.0 Hz, 2H), 1.50–1.37 (m, 2H), 1.22–1.11 (m, 2H), and 0.83 (t, *J* = 7.3 Hz, 3H).

According to **GP2** (method B), starting from the isolated compound, **compound BA-115** was isolated as a white solid (44 mg, 0.0939 mmol, 97%).

C23H24ClN3O4S; MS (ESI+) m/z: 474 [M + H]+; 1H NMR (400 MHz, DMSO-d6) δ 13.19 (s, 1H), 7.83 (d, *J* = 1.9 Hz, 1H), 7.43 (d, *J* = 2.0 Hz, 1H), 7.23 (dd, *J* = 8.6, 7.2 Hz, 2H), 7.18 (s, 1H), 7.05–6.98 (m, 3H), 6.81 (dd, *J* = 8.1, 1.4 Hz, 2H), 6.48 (d, *J* = 8.0 Hz, 2H), 5.09–5.03 (m, 1H), 3.07 (q, *J* = 6.6 Hz, 2H), 1.42–1.33 (m, 2H), 1.18–1.07 (m, 2H), and 0.78 (t, *J* = 7.3 Hz, 3H). 
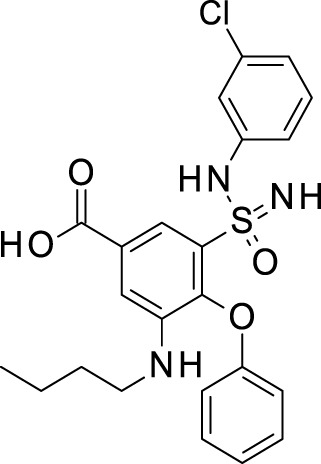


3-(butylamino)-5-[(3-chloroanilino)sulfonimidoyl]-4-phenoxy-benzoic acid (**compound BA-100**).

According to **GP1** (method B), starting from **Int02** and 3-chloroaniline, an off-white foam was isolated (224 mg, 0.4590 mmol, 91%).

C24H26ClN3O4S; MS (ESI+) m/z: 488 [M + H]+; 1H NMR (400 MHz, chloroform-d): δ 8.09 (d, *J* = 2.0 Hz, 1H), 7.59 (d, *J* = 2.0 Hz, 1H), 7.35–7.28 (m, 2H), 7.17–7.11 (m, 1H), 7.02–6.96 (m, 1H), 6.91–6.83 (m, 3H), 6.69–6.64 (m, 1H), 6.56 (t, *J* = 2.1 Hz, 1H), 3.95 (s, 3H), 3.12 (t, *J* = 7.0 Hz, 2H), 1.48–1.39 (m, 2H), 1.23–1.13 (m, 2H), and 0.83 (t, *J* = 7.3 Hz, 3H).

According to **GP2** (method A), starting from the intermediate, **compound BA-100** was isolated as an off-white foam (221 mg, 0.4476 mmol, 98%).

C23H24ClN3O4S; MS (ESI+) m/z: 474 [M + H]+; 1H NMR (400 MHz, DMSO-d6): δ 13.14 (s, 1H), 7.83 (d, *J* = 1.9 Hz, 1H), 7.44 (d, *J* = 2.0 Hz, 1H), 7.23 (dd, *J* = 8.6, 7.3 Hz, 2H), 7.01 (q, *J* = 7.8 Hz, 2H), 6.83–6.79 (m, 2H), 6.76 (dd, *J* = 7.8, 2.1 Hz, 1H), 6.47 (d, *J* = 8.1 Hz, 1H), 6.37 (s, 1H), 5.13 (t, *J* = 5.7 Hz, 1H), 3.08 (q, *J* = 6.4 Hz, 2H), 1.42–1.33 (m, 2H), 1.19–1.08 (m, 2H), and 0.78 (t, *J* = 7.4 Hz, 3H).

13C NMR (101 MHz, DMSO-d6): δ 167, 157 (2C), 143, 140, 138, 133, 130, 129 (2C), 128, 122 (2C), 121, 120, 116 (3C), 115, 42.4, 30.5, 19.7, and 14.0.
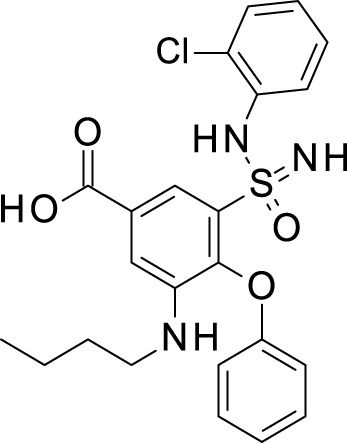


3-(butylamino)-5-[(2-chloroanilino)sulfonimidoyl]-4-phenoxy-benzoic acid (**compound BA-114**).

According to **GP1** (method B), starting from **Int02** and 2-chloroaniline, a white solid was isolated (63 mg, 0.1291 mmol, 28%).

C24H26ClN3O4S; MS (ESI+) m/z: 488 [M + H]+; 1H NMR (400 MHz, chloroform-d): δ 8.26 (d, *J* = 1.9 Hz, 1H), 7.56 (d, *J* = 2.0 Hz, 1H), 7.34 (dd, *J* = 8.1, 1.5 Hz, 1H), 7.30–7.26 (m, 3H), 7.08 (td, *J* = 7.7, 1.6 Hz, 2H), 6.93–6.89 (m, 3H), 3.95 (s, 3H), 3.09 (t, *J* = 6.9 Hz, 2H), 1.46–1.34 (m, 2H), 1.21–1.06 (m, 2H), and 0.81 (t, *J* = 7.3 Hz, 3H).

According to **GP2** (method B), starting from the compound isolated, **compound BA-114** was isolated as a white solid (31 mg, 0.0646 mmol, 96%).

C23H24ClN3O4S; MS (ESI+) m/z: 474 [M + H]+; 1H NMR (400 MHz, DMSO-d6): δ 13.13 (s, 1H), 8.07 (d, *J* = 1.9 Hz, 1H), 7.42 (d, *J* = 1.9 Hz, 1H), 7.26–7.22 (m, 3H), 7.22–7.19 (m, 2H), 7.08–6.95 (m, 2H), 6.85–6.75 (m, 3H), 4.93 (t, *J* = 5.7 Hz, 1H), 3.05 (q, *J* = 6.5 Hz, 2H), 1.40–1.29 (m, 2H), 1.14–1.03 (m, 2H), and 0.76 (t, *J* = 7.3 Hz, 3H).

13C NMR (101 MHz, DMSO-d6): δ 166.60, 156.04, 142.35, 139.94, 137.62, 129.40, 128.99 (2C), 127.44, 126.83 (2C), 122.59 (2C), 122.19, 121.30, 116.61, 115.63 (2C), 114.75, 42.03, 30.12, 19.25, and 13.56.
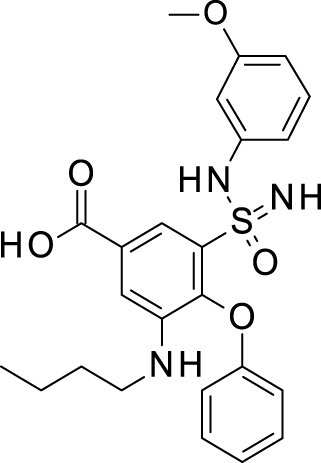


3-(butylamino)-5-[(3-methoxyanilino)sulfonimidoyl]-4-phenoxy-benzoic acid (**compound 84**).

According to **GP1** (method A), starting from **Int02** and 3-methoxyaniline, a white solid was isolated (151 mg, 0.3091 mmol, 64%).

C25H29N3O5S; MS (ESI+) m/z: 484 [M + H]+; 1H NMR (400 MHz, chloroform-d): δ 8.11 (d, *J* = 2.0 Hz, 1H), 7.56 (d, *J* = 2.0 Hz, 1H), 7.33–7.27 (m, 2H), 7.13–7.07 (m, 1H), 6.99 (t, *J* = 8.1 Hz, 1H), 6.93–6.87 (m, 2H), 6.46 (dddd, *J* = 11.8, 7.9, 2.2, 0.9 Hz, 2H), 6.23 (t, *J* = 2.2 Hz, 1H), 3.94 (s, 3H), 3.64 (s, 3H), 3.11 (t, *J* = 6.9 Hz, 2H), 1.47–1.37 (m, 2H), 1.16 (h, *J* = 7.4 Hz, 2H), and 0.82 (t, *J* = 7.3 Hz, 3H).

According to **GP2** (method A), starting from the isolated compound, **compound BA-84** was isolated as an off-white solid (116 mg, 0.2470 mmol, 79%).

C24H27N3O5S; MS (ESI+) m/z: 470 [M + H]+; 1H NMR (400 MHz, DMSO-d6): δ 13.11 (s, 1H), 7.85 (d, *J* = 2.0 Hz, 1H), 7.42 (d, *J* = 2.0 Hz, 1H), 7.26–7.21 (m, 2H), 7.03–6.97 (m, 1H), 6.89 (t, *J* = 8.1 Hz, 1H), 6.84–6.81 (m, 2H), 6.32 (dd, *J* = 8.1, 2.5 Hz, 1H), 6.28–6.23 (m, 1H), 6.01 (s, 1H), 5.02 (s, 1H), 3.55 (s, 3H), 3.06 (t, *J* = 6.8 Hz, 2H), 1.43–1.31 (m, 2H), 1.18–1.07 (m, 2H), and 0.78 (t, *J* = 7.3 Hz, 3H).

13C NMR (101 MHz, DMSO-d6): δ 167, 160, 156, 143, 140, 129 (4C), 128, 123, 116 (3C), 115 (2C), 109, 107 (2C), 55.1, 42.4, 30.6, 19.7, and 14.1.
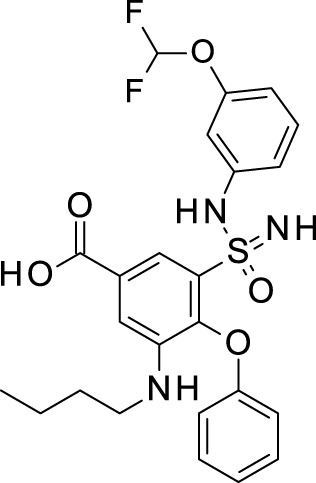


3-(butylamino)-5-[[3-(difluoromethoxy)anilino]sulfonimidoyl]-4-phenoxy-benzoic acid (**compound BA-111**).

According to **GP1** (method B), starting from **Int02** and 3-(difluoromethoxy)aniline, a white solid was isolated (209 mg, 0.4023 mmol, 87%).

C25H27F2N3O5S; MS (ESI+) m/z: 520 [M + H]+; 1H NMR (400 MHz, chloroform-d): δ 8.09 (d, J = 2.0 Hz, 1H), 7.59 (d, J = 2.0 Hz, 1H), 7.31 (dd, J = 8.5, 7.2 Hz, 2H), 7.17–7.09 (m, 1H), 7.05 (t, J = 8.1 Hz, 1H), 6.92–6.85 (m, 2H), 6.71–6.61 (m, 2H), 6.43 (d, J = 74.4 Hz, 1H), 6.31 (t, J = 2.3 Hz, 1H), 3.95 (s, 3H), 3.12 (t, J = 7.0 Hz, 2H), 1.49–1.37 (m, 2H), 1.24–1.12 (m, 2H), and 0.83 (t, J = 7.3 Hz, 3H).

According to GP2 (method B), starting from the isolated compound, compound BA-111 was isolated as a white solid (25 mg, 0.0472 mmol, 52%).

C24H25F2N3O5S; MS (ESI+) m/z: 506 [M + H]+; 1H NMR (400 MHz, DMSO-d6): δ 13.15 (s, 1H), 7.84 (d, *J* = 2.0 Hz, 1H), 7.43 (d, *J* = 2.0 Hz, 1H), 7.26–7.19 (m, 3H), 7.04–6.99 (m, 2H), 6.83–6.80 (m, 2H), 6.54 (dd, *J* = 8.2, 2.4 Hz, 1H), 6.44 (d, *J* = 8.0 Hz, 1H), 6.21 (s, 1H), 5.09 (t, *J* = 5.8 Hz, 1H), 3.07 (q, *J* = 6.5 Hz, 2H), 1.42–1.33 (m, 2H), 1.19–1.07 (m, 2H), and 0.78 (t, *J* = 7.3 Hz, 3H).

13C NMR (101 MHz, DMSO-d6): δ 166.55 (2C), 156.01 (2C), 142.64 (2C), 139.50, 129.31, 128.86 (2C), 122.01 (2C), 121.80, 117.86, 116.32, 115.58 (2C), 114.69, 113.33, 110.33, 42.00, 30.13, 19.30, and 13.58.
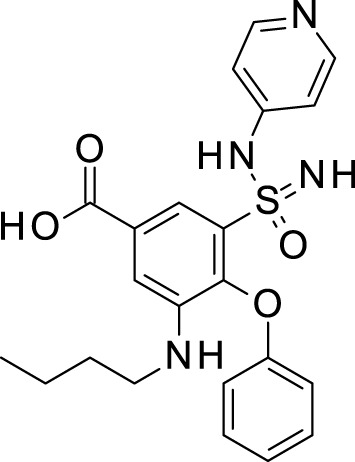


3-(butylamino)-4-phenoxy-5-[(4-pyridylamino)sulfonimidoyl]benzoic acid (**compound BA-164**).

According to **GP1** (method B), starting from **Int02** and pyridine-4-amine, a white solid was isolated (10 mg, 0.0418 mmol, 10%).

C23H26N4O4S; MS (ESI+) m/z: 455 [M + H]+; 1H NMR (400 MHz, chloroform-d) δ 8.16 (d, *J* = 5.4 Hz, 2H), 8.09 (d, *J* = 2.0 Hz, 1H), 7.60 (d, *J* = 2.0 Hz, 1H), 7.32–7.27 (m, 2H), 7.15–7.10 (m, 1H), 6.85–6.81 (m, 2H), 6.54 (d, *J* = 5.4 Hz, 2H), 3.96 (s, 3H), 3.90 (t, *J* = 5.4 Hz, 1H), 3.12 (q, *J* = 6.6 Hz, 2H), 1.49–1.39 (m, 2H), 1.24–1.11 (m, 2H), and 0.83 (t, *J* = 7.3 Hz, 3H).

According to **GP2** (method A), starting from the isolated compound, **compound BA-164** was isolated as a white solid (16 mg, 0.0374 mmol, 96%).

C22H24N4O4S; MS (ESI+) m/z: 441 [M + H]+; 1H NMR (400 MHz, DMSO-d6) δ 8.08 (d, *J* = 5.8 Hz, 2H), 7.84 (d, *J* = 1.9 Hz, 1H), 7.47 (d, *J* = 1.9 Hz, 1H), 7.19 (t, *J* = 7.8 Hz, 2H), 6.99 (t, *J* = 7.3 Hz, 1H), 6.75 (d, *J* = 8.1 Hz, 2H), 6.48 (d, *J* = 5.8 Hz, 2H), 5.25 (t, *J* = 5.7 Hz, 1H), 3.07 (q, *J* = 6.6 Hz, 2H), 1.44–1.31 (m, 2H), 1.19–1.06 (m, 2H), and 0.78 (t, *J* = 7.3 Hz, 3H).
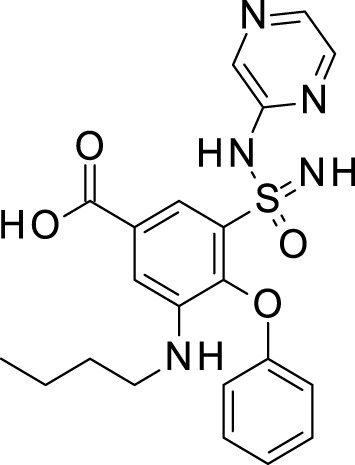


3-(butylamino)-4-phenoxy-5-[(pyrazin-2-ylamino)sulfonimidoyl]benzoic acid (**compound BA-104**).

According to **GP1** (method A), starting from **Int02** and pyrazin-2-amine, a yellow oil was isolated (30 mg, 0.0667 mmol, 13%).

C22H25N5O4S; MS (ESI+) m/z: 456 [M + H]+; 1H NMR (400 MHz, chloroform-d): δ 8.06 (d, *J* = 1.9 Hz, 1H), 7.94–7.91 (m, 2H), 7.59 (d, *J* = 1.2 Hz, 1H), 7.57 (d, *J* = 2.0 Hz, 1H), 7.24–7.18 (m, 2H), 7.09–7.04 (m, 1H), 6.75–6.71 (m, 2H), 5.80 (s, 1H), 3.94 (s, 3H), 3.87 (t, *J* = 5.3 Hz, 1H), 3.15–3.06 (m, 2H), 1.47–1.37 (m, 2H), 1.22–1.11 (m, 2H), and 0.82 (t, *J* = 7.3 Hz, 3H).

According to **GP2** (method A), starting from the isolated compound, **compound BA-104** was isolated as a brown foam (20 mg, 0.0453 mmol, 10%).

C21H23N5O4S; MS (ESI+) m/z: 442 [M + H]+; 1H NMR (400 MHz, DMSO-d6): δ 13.19 (s, 1H), 7.87 (d, *J* = 2.0 Hz, 1H), 7.83–7.81 (m, 1H), 7.75 (d, *J* = 2.7 Hz, 1H), 7.43–7.40 (m, 2H), 7.11 (d, *J* = 1.5 Hz, 1H), 7.08–7.02 (m, 2H), 6.88 (t, *J* = 7.3 Hz, 1H), 6.62–6.58 (m, 2H), 5.06 (t, *J* = 6.0 Hz, 1H), 3.05 (q, *J* = 6.3 Hz, 2H), 1.40–1.30 (m, 2H), 1.16–1.06 (m, 2H), and 0.77 (t, *J* = 7.3 Hz, 3H).

13C NMR (101 MHz, DMSO-d6): δ 167, 156 (2C), 155, 142, 141, 139, 135, 134, 129 (2C), 122, 118, 116 (2C), 115 (2C), 42.4, 30.4, 19.7, and 14.0.
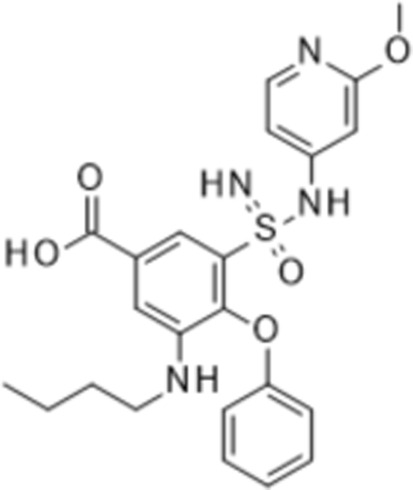


3-(butylamino)-5-[[(2-methoxy-4-pyridyl)amino]sulfonimidoyl]-4-phenoxy-benzoic acid (**compound BA-193**).

According to **GP1** (method A), starting from **Int02** and 2-methoxypyridin-4-amine, a white solid was isolated (31 mg, 0.064 mmol, 13%).

C24H28N4O5S; MS (ESI+) m/z: 485 [M + H]+; 1H NMR (400 MHz, chloroform-d): δ 8.09 (d, J = 1.9 Hz, 1H), 7.78 (d, J = 5.7 Hz, 1H), 7.61 (d, J = 1.9 Hz, 1H), 7.35–7.28 (m, 2H), 7.14 (t, J = 7.4 Hz, 1H), 6.86 (d, J = 8.0 Hz, 2H), 6.23 (dd, J = 5.7, 1.8 Hz, 1H), 6.04 (d, J = 1.8 Hz, 1H), 4.01–3.94 (m, 3H), 3.91 (t, J = 5.5 Hz, 1H), 3.83 (s, 3H), 3.14 (q, J = 6.5 Hz, 2H), 1.51–1.40 (m, 2H), 1.26–1.15 (m, 2H), and 0.85 (t, J = 7.3 Hz, 3H).

According to **GP2** (method A), starting from the isolated compound, **compound BA-193** was isolated as a white solid (24 mg, 0.0510 mmol, 80%).

C23H26N4O5S; MS (ESI+) m/z: 471 [M + H]+; 1H NMR (400 MHz, DMSO-d_6_): 3.13 (s, 1H), 7.83 (d, J = 1.9 Hz, 1H), 7.65 (d, J = 5.7 Hz, 1H), 7.45 (d, J = 2.0 Hz, 3H), 7.25–7.17 (m, 2H), 7.01 (t, J = 7.3 Hz, 1H), 6.79 (d, J = 8.0 Hz, 2H), 6.05 (d, J = 5.6 Hz, 1H), 5.82 (s, 1H), 5.12 (t, J = 5.8 Hz, 1H), 3.69 (s, 3H), 3.08 (q, J = 6.6 Hz, 2H), 1.42–1.35 (m, 2H), 1.16–1.08 (m, 2H), and 0.78 (t, J = 7.3 Hz, 3H).

13C NMR (101 MHz, DMSO) δ 166.98, 164.81, 156.49, 155.02, 146.52, 143.24, 139.99, 137.53, 129.35 (2C), 122.58, 116.26, 116.04 (2C), 115.45, 112.92, 101.75, 53.16, 42.49, 30.62, 19.79, and 14.06.
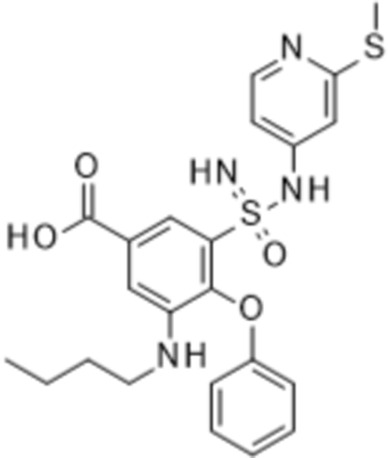


3-(butylamino)-5-[[(2-methylsulfanyl-4-pyridyl)amino]sulfonimidoyl]-4-phenoxy-benzoic acid (**compound BA-192**).

According to **GP1** (method A), starting from **Int02** and 2-(Methylthio)pyridin-4-amine, a white solid was isolated (45 mg, 0.0899 mmol, 18%).

C24H28N4O4S2; MS (ESI+) m/z: 501 [M + H]+; 1H NMR (400 MHz, chloroform-d): δ 8.09 (d, J = 2.0 Hz, 1H), 8.02 (d, J = 5.6 Hz, 1H), 7.61 (d, J = 2.0 Hz, 1H), 7.35–7.29 (m, 2H), 7.19–7.04 (m, 1H), 6.84 (d, J = 8.1 Hz, 2H), 6.37 (dd, J = 5.6, 2.0 Hz, 1H), 6.32 (d, J = 2.0 Hz, 1H), 3.98 (s, 3H), 3.92 (t, J = 5.5 Hz, 1H), 3.14 (q, J = 6.6 Hz, 2H), 2.44 (s, 3H), 1.52–1.42 (m, 2H), 1.27–1.14 (m, 2H), and 0.85 (t, J = 7.3 Hz, 3H).

According to **GP2** (method A), starting from the isolated compound, **compound BA-192** was isolated as a white solid (38 mg, 0.0781 mmol, 87%).

C23H26N4O4S2; MS (ESI+) m/z: 487 [M + H]+; 1H NMR (400 MHz, DMSO-d_6_): δ 7.93 (d, J = 5.8 Hz, 1H), 7.83 (d, J = 1.9 Hz, 1H), 7.68 (s, 2H), 7.47 (d, J = 1.9 Hz, 1H), 7.22 (t, J = 7.8 Hz, 2H), 7.01 (t, J = 7.3 Hz, 1H), 6.77 (d, J = 8.1 Hz, 2H), 6.34–6.20 (m, 1H), 6.14 (s, 1H), 5.22 (s, 1H), 3.09 (q, J = 6.5 Hz, 2H), 2.36 (s, 3H), 1.42–1.36 (m, 2H), 1.18–1.09 (m, 2H), and 0.79 (t, J = 7.4 Hz, 3H).

13C NMR (101 MHz, DMSO) δ 166.90, 158.45, 156.48, 154.21, 147.90, 143.37, 139.87, 137.08, 129.35 (2C), 128.58, 122.61, 116.16, 115.99 (2C), 115.68, 113.99, 113.42, 42.47, 30.61, 19.81, 14.06, and 13.35.
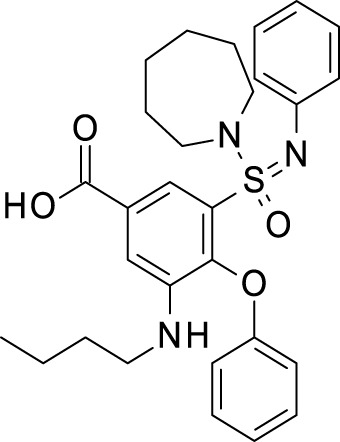


3-[S-(azepan-1-yl)-N-phenyl-sulfonimidoyl]-5-(butylamino)-4-phenoxy-benzoic acid (**compound BA-103**).

In a sealed tube, copper acetate (II) (49 mg, 0.272 mmol, 1.25 eq.), triethylamine (0.040 mL, 0.2720 mmol, 1.25 eq.), and phenylboronic acid (40 mg, 0.326 mmol, 1.5 eq.) were added successively to a solution of c**ompound BA-70-E** (100 mg, 0.217 mmol, 1.00 eq.) in acetonitrile (0.7860 mL, 0.5 M) under an O_2_ atmosphere. The reaction mixture was stirred at 20 °C for 24 h. The conversion was checked by LC–MS. The reaction mixture was concentrated under reduced pressure. The crude residue was purified by automated flash chromatography with cyclohexane/EtOAc (gradient from 99/1 to 65/35) to obtain a brown oil (m = 8 mg, 0.0149 mmol, 7%).

According to **GP2** (method A), starting from the isolated compound, **compound BA-103** was isolated as a colorless oil (7 mg, 0.0134 mmol, 90%).

C29H35N3O4S; MS (ESI+) m/z: 522 [M + H]+; 1H NMR (400 MHz, acetonitrile-d3): δ 8.03 (d, *J* = 2.0 Hz, 1H), 7.51 (d, *J* = 2.0 Hz, 1H), 7.36–7.27 (m, 2H), 7.17–7.03 (m, 3H), 6.92–6.78 (m, 5H), 4.40–4.22 (m, 1H), 3.42–3.23 (m, 4H), 3.10 (t, *J* = 7.0 Hz, 2H), 1.58–1.30 (m, 10H), 1.18–1.07 (m, 2H), and 0.80 (t, *J* = 7.4 Hz, 3H).

13C NMR (101 MHz, acetonitrile-d3): δ 166.6, 156.8, 145.1, 143.7, 130.0 (2C), 129.3 (2C), 127.9, 124.0 (2C), 123.1, 121.9, 119.2, 116.0 (3C), 49.6 (2C), 43.1, 31.1, 29.3 (2C), 27.0 (2C), 20.1, and 13.5. (+2C overlapping with the CD3CN signal.)
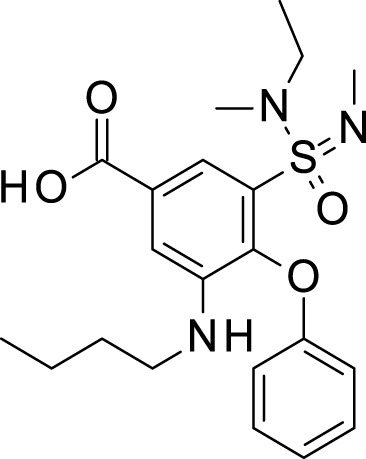


3-(butylamino)-5-[S-[ethyl(methyl)amino]-N-methyl-sulfonimidoyl]-4-phenoxybenzoic acid (**compound BA-67**).

In a sealed tube, copper acetate (II) (30 mg, 0.164 mmol, 1.50 eq.), pyridine (0.021 mL, 0.263 mmol, 2.40 eq.), and methylboronic acid (13 mg, 0.219 mmol, 2.00 eq.) were added successively to a solution of **compound BA-66-E** (50 mg, 0.110 mmol, 1.00 eq.) in 1,4-dioxane (0.5482 mL, 0.2 M) under an O_2_ atmosphere. The reaction mixture was stirred at 100 °C for 16 h. The conversion was checked by LC–MS. The reaction mixture was concentrated under reduced pressure. The crude residue was purified by automated flash chromatography with cyclohexane/EtOAc (gradient from 99/1 to 65/35) to obtain a colorless oil (m = 6 mg, 0.0138 mmol, 13%).

C22H31N3O4S; MS (ESI+) m/z: 434 [M + H]+; 1H NMR (400 MHz, chloroform-d): δ 8.05 (d, *J* = 2.0 Hz, 1H), 7.48 (d, *J* = 2.0 Hz, 1H), 7.32–7.22 (m, 2H), 7.08–6.98 (m, 1H), 6.87–6.79 (m, 2H), 3.93 (s, 3H), 3.89–3.83 m, 1H), 3.32–3.19 (m, 1H), 3.17–3.03 (m, 3H), 2.77 (s, 3H), 2.67 (s, 3H), 1.47–1.35 (m, 2H), 1.21–1.12 (m, 2H), 1.10 (t, *J* = 7.1 Hz, 3H), and 0.81 (t, *J* = 7.3 Hz, 3H).

According to **GP2** (method A), starting from the isolated compound, **compound BA-67**, a colorless sticky oil, was isolated (5.6 mg, 0.0129 mmol, 94%).

C21H29N3O4S; MS (ESI+) m/z: 420 [M + H]+; 1H NMR (400 MHz, DMSO-d6): δ 13.10 (s, 1H), 7.88 (d, *J* = 2.0 Hz, 1H), 7.39 (d, *J* = 2.1 Hz, 1H), 7.31–7.23 (m, 2H), 7.06–6.97 (m, 1H), 6.79–6.73 (m, 2H), 4.96–4.88 (m, 1H), 3.20–3.08 (m, 1H), 3.09–2.95 (m, 3H), 2.67 (s, 3H), 2.52 (s, 3H), 1.40–1.29 (m, 2H), 1.15–1.05 (m, 2H), 1.01 (t, *J* = 7.1 Hz, 3H), and 0.76 (t, *J* = 7.3 Hz, 3H).

13C NMR (101 MHz, DMSO-d6): δ 166.6, 156.1, 142.5, 139.9, 133.2, 129.2 (2C), 127.8, 122.1, 118.6, 115.2 (2C), 114.4, 44.4, 42, 33.9, 30.1, 27.6, 19.3, 13.6, and 13.2.
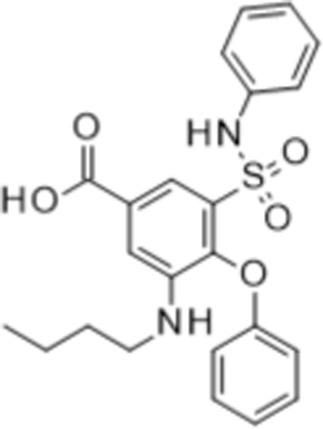


3-(butylamino)-4-phenoxy-5-(phenylsulfamoyl)benzoic acid (**compound BA-83**).

According to **GP4**, starting from **Int01** and benzylbromide, a white solid was isolated (1.45 g, 3.2 mmol, 97%).

C24H26N2O5S; MS (ESI+) m/z: 455 [M + H]+; 1H NMR (400 MHz, DMSO-d6): δ 10.24 (s, 1H), 7.69 (d, J = 2.0 Hz, 1H), 7.39 (d, J = 2.0 Hz, 1H), 7.32–7.16 (m, 4H), 7.08–6.97 (m, 4H), 6.82–6.77 (m, 2H), 5.10 (t, J = 5.6 Hz, 1H), 3.86 (s, 3H), 3.00 (q, J = 6.5 Hz, 2H), 1.36–1.24 (m, 2H), 1.09–0.98 (m, 2H), and 0.73 (t, J = 7.3 Hz, 3H).

According to **GP2** (method A), starting from the isolated compound, **compound BA-83** was isolated as a white solid (1.3 g, 2.95 mmol, 92%).

C23H24N2O5S; MS (ESI+) m/z: 441 [M + H]+; 1H NMR (400 MHz, DMSO-d6): δ 13.21 (s, 1H), 10.21 (s, 1H), 7.69 (d, *J* = 2.0 Hz, 1H), 7.40 (d, *J* = 2.0 Hz, 1H), 7.28–7.18 (m, 4H), 7.08–6.97 (m, 4H), 6.82–6.78 (m, 2H), 5.03 (t, *J* = 5.7 Hz, 1H), 3.00 (q, *J* = 6.5 Hz, 2H), 1.37–1.26 (m, 2H), 1.09–0.98 (m, 2H), and 0.73 (t, *J* = 7.3 Hz, 3H).

13C NMR (101 MHz, DMSO-d6): δ 166.8, 156.5, 142.9, 140.5, 138.1, 134.1, 129.6 (2C), 129.5 (2C), 128.8, 124.0, 122.9, 119.7 (2C), 117.2, 116.2, 116.2 (2C), 42.4, 30.5, 19.7, and 14.0.
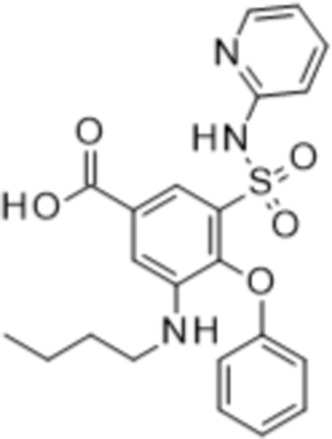


3-(butylamino)-4-phenoxy-5-(2-pyridylsulfamoyl)benzoic acid (**compound BA-189**).

According to **GP4**, starting from **Int01** and 2-bromopyridine, a white solid was isolated (142 mg, 0.3117 mmol, 59%).

C23H25N3O5S; MS (ESI+) m/z: 456 [M + H]+; 1H NMR (400 MHz, DMSO-d6): δ 7.83 (d, *J* = 2.0 Hz, 1H), 7.77 (s, 1H), 7.46 (s, 1H), 7.30 (s, 1H), 7.14 (t, *J* = 7.8 Hz, 2H), 6.92 (t, *J* = 7.3 Hz, 1H), 6.81 (s, 1H), 6.67 (d, *J* = 8.0 Hz, 2H), 6.59 (s, 1H), 4.81 (s, 1H), 3.87 (s, 3H), 3.00 (q, *J* = 6.4 Hz, 2H), 1.36–1.32 (m, 2H), 1.08–1.03 (m, 2H), and 0.74 (t, *J* = 7.3 Hz, 3H).

According to **GP2** (method A), starting from the isolated compound, **compound Ba-189** was isolated as a white solid (128 mg, 0.2899 mmol, 93%).

C22H23N3O5S; MS (ESI+) m/z: 442 [M + H]+; 1H NMR (400 MHz, DMSO-d6): δ 12.70 (s, 1H), 7.82 (s, 1H), 7.76 (s, 1H), 7.66 (t, J = 8.0 Hz, 1H), 7.38 (s, 1H), 7.14 (t, J = 7.7 Hz, 2H), 7.03 (d, J = 8.9 Hz, 1H), 6.93 (t, J = 7.4 Hz, 1H), 6.81–6.70 (m, 1H), 6.63 (d, J = 8.0 Hz, 2H), 4.91 (s, 1H), 3.10–2.87 (m, 2H), 1.34–1.32 (m, 2H), 1.11–1.03 (m, 2H), and 0.74 (t, J = 7.4 Hz, 3H).

13C NMR (101 MHz, DMSO) δ 167.07, 156.58, 156.49, 154.47, 142.78, 141.57, 140.37, 137.30, 129.44 (2C), 128.45, 122.42, 117.03, 115.72 (3C), 115.11, 113.98, 42.49, 30.58, 19.72, and 14.03.
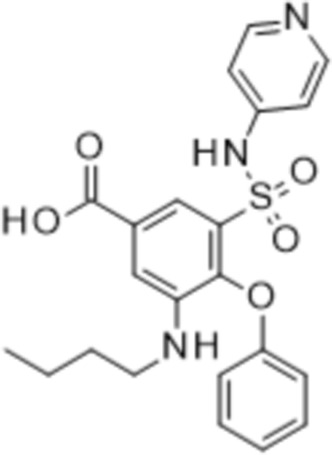


3-(butylamino)-4-phenoxy-5-(4-pyridylsulfamoyl)benzoic acid (**compound BA-190)**.

According to **GP4**, starting from **Int01** and 4-bromopyridine, a white solid was isolated as (160 mg, 0.3512 mmol, 66%).

C23H25N3O5S; MS (ESI+) m/z: 456 [M + H]+; 1H NMR (400 MHz, DMSO-d6): δ 12.57 (s, 1H), 787–7.77 (m, 3H), 7.35 (s, 1H), 7.15 (d, J = 7.9 Hz, 2H), 6.94 (s, 1H), 6.61 (s, 4H), 4.94 (s, 1H), 3.89 (s, 3H), 3.02 (q, J = 6.4 Hz, 2H), 1.37–1.28 (m, 2H), 1.11–1.06 (m, 2H), and 0.75 (t, J = 7.4 Hz, 3H)

According to **GP2** (method A), starting from the isolated compound, **compound BA-190** was isolated as a white solid (140 mg, 0.3171 mmol, 90%).

C22H23N3O5S; MS (ESI+) m/z: 442 [M + H]+; 1H NMR (400 MHz, DMSO-d6): δ 12.70 (s, 1H), 7.83 (d, J = 32.3 Hz, 3H), 7.36 (s, 1H), 7.16 (t, J = 7.7 Hz, 2H), 6.94 (t, J = 7.3 Hz, 1H), 6.65 (d, J = 7.7 Hz, 4H), 5.03–4.72 (m, 1H), 3.01 (q, J = 6.5 Hz, 2H), 1.37–1.32 (m, 2H), 1.11–1.06 (m, 2H), and 0.75 (t, J = 7.3 Hz, 3H).

13C NMR (101 MHz, DMSO) δ 167.29, 165.63, 156.79, 142.80, 140.29, 129.34 (2C), 128.81, 122.27, 116.86, 115.88 (2C), 114.76 (2C), 42.56, 30.67, 19.75, and 14.03.

3-(butylamino)-5-cyano-2-phenoxy-N-phenyl-benzenesulfonamide (**Int15**).

CDI (101 mg, 0.622 mmol, 1.1 equiv.) was added to a stirred solution of **compound BA-83** (283 mg, 0.565 mmol, 1 equiv.) in CH_3_CN (5.6 mL, 0.1 M). The resulting mixture was stirred at 50 °C for 1 h. A 28% aqueous solution of ammonium hydroxide (90 µL, 0.622 mmol, 1.1 equiv.) was added after cooling at 20 °C. The reaction mixture was stirred at 20 °C for 16 h. The reaction mixture was concentrated under reduced pressure, and the crude residue was purified by automated flash chromatography with DCM/MeOH (gradient from 100/0 to 90/10 over 13 CV) to obtain a yellow oil (234 mg, 0.533 mmol, 94%).

C23H25N3O4S; MS (ESI+) m/z: 440 [M + H]+; 1H NMR (400 MHz, DMSO-d6): δ 10.13 (s, 1H), 8.12 (s, 1H), 7.67 (d, *J* = 2.1 Hz, 1H), 7.43 (s, 1H), 7.39 (d, *J* = 2.0 Hz, 1H), 7.29–7.15 (m, 4H), 7.08–6.93 (m, 4H), 6.84–6.76 (m, 2H), 4.83 (t, *J* = 5.7 Hz, 1H), 3.01 (q, *J* = 6.6 Hz, 2H), 1.37–1.23 (m, 2H), 1.15–0.98 (m, 2H), and 0.73 (t, *J* = 7.3 Hz, 3H).

Dry pyridine (25 µL, 0.315 mmol, 1 equiv.) and TFAA (44 µL, 0.315 mmol, 1 equiv.) were added to a stirred solution of the compound isolated (180 mg, 0.315 mmol, 1 equiv.) in CH_3_CN (1.8 mL, 0.18 M). The resulting mixture was stirred at 20 °C for 4 h. The reaction mixture was concentrated under reduced pressure, and the crude residue was purified by automated flash chromatography with cyclohexane/EtOAc (gradient from 100/0 to 40/60 over 10 CV) to obtain **Int15** (135 mg, 0.266 mmol, 84%) as a colorless oil.

C23H23N3O3S; MS (ESI+) m/z: 422 [M + H]+; 1H NMR (400 MHz, DMSO-d6): δ 10.31 (s, 1H), 7.41–7.35 (m, 2H), 7.29–7.20 (m, 4H), 7.08–7.00 (m, 4H), 6.82–6.77 (m, 2H), 5.35 (t, *J* = 5.7 Hz, 1H), 3.00 (q, *J* = 6.6 Hz, 2H), 1.33–1.24 (m, 2H), 1.08–0.96 (m, 2H), and 0.72 (t, *J* = 7.3 Hz, 3H).
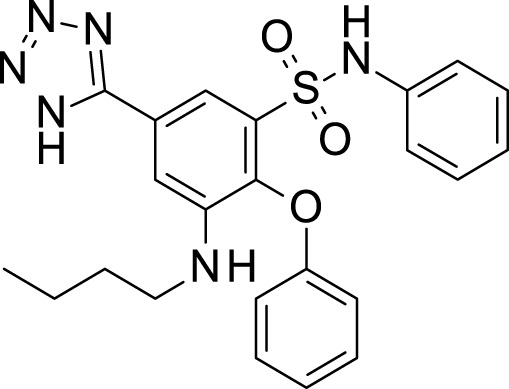


3-(butylamino)-2-phenoxy-N-phenyl-5-(1H-tetrazol-5-yl)benzenesulfonamide (**compound BA-172**).

Sodium azide (32 mg, 0.498 mmol, 4.5 equiv.) and ammonium chloride (25 mg, 0.468 mmol, 3.9 equiv.) were added to a stirred solution of **Int15** (50 mg, 0.120 mmol, 1 equiv.) in DMF (2 mL, 0.06 M). The resulting mixture was stirred at 120 °C for 4 h. After cooling at 20 °C, the reaction mixture was diluted with EtOAc and washed with brine. The organic layer was dried over Na_2_SO_4_, filtered, and concentrated under reduced pressure to obtain **compound BA-172** (51 mg, 0.110 mmol, 91%) as an off-white solid.

C23H24N6O3S; MS (ESI+) m/z: 465 [M + H]+; 1H NMR (400 MHz, DMSO-d6): δ 10.10 (s, 1H), 7.81 (d, *J* = 1.8 Hz, 1H), 7.53 (d, *J* = 1.9 Hz, 1H), 7.28–7.15 (m, 5H), 7.10–7.05 (m, 2H), 7.03–6.93 (m, 2H), 6.85–6.81 (m, 2H), 4.75 (t, *J* = 5.7 Hz, 1H), 3.02 (q, *J* = 6.6 Hz, 2H), 1.39–1.30 (m, 2H), 1.14–1.03 (m, 2H), and 0.75 (t, *J* = 7.3 Hz, 3H).

13C NMR (101 MHz, DMSO-d6): δ 159.20, 156.68, 142.21, 137.97, 136.04, 133.48, 128.97 (2C), 128.87 (3C), 123.15, 121.99, 118.92 (2C), 115.62 (2C), 113.53, 112.74, 42.14, 30.27, 19.28, and 13.58.
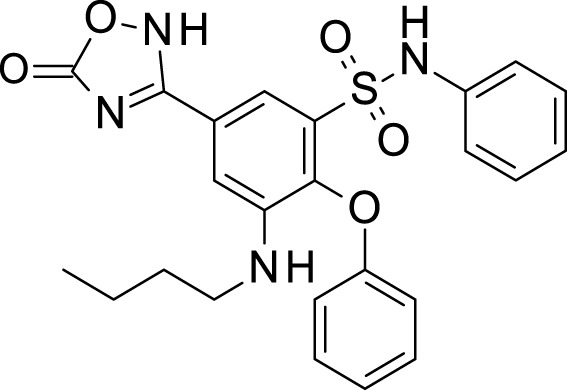


3-(butylamino)-5-(5-oxo-2H-1,2,4-oxadiazol-3-yl)-2-phenoxy-N-phenyl-benzenesulfonamide (**compound 171**).

A solution of hydroxylamine hydrochloride (37 mg, 0.532 mmol, 2 equiv.) in a 1M aqueous solution of NaHCO3 (800 µL, 3 equiv.) was added to a stirred solution of **Int15** (135 mg, 0.266 mmol, 1 equiv.) in EtOH (3 mL, 0.09 M), and the resulting white suspension was stirred at 80 °C for 4 h. The reaction mixture was cooled down to 20 °C and poured into water. The organic layer was extracted twice with EtOAc. The combined organic layers were concentrated under reduced pressure to obtain an off-white solid (116 mg, 0.255 mmol, 96%).

C23H26N4O4S; MS (ESI+) m/z: 455 [M + H]+.

DBU (42 µL, 0.280 mmol, 1.10 equiv.) and CDI (58 mg, 0.383 mg, 1.5 eq.) were added to a stirred solution of the isolated compound (116 mg, 0.255 mmol, 1 equiv.) in dioxane (2.5 mL, 0.1 M). The resulting solution was stirred at 100 °C for 16 h. The reaction mixture was concentrated under reduced pressure, and the crude residue was purified by automated flash chromatography with DCM/MeOH (gradient from 100/0 to 90/10 over 10 CV) to obtain **compound BA-171** (62 mg, 0.129 mmol, 51%) as a yellow oil.

C24H24N4O5S; MS (ESI+) m/z: 481 [M + H]+; 1H NMR (400 MHz, DMSO-d6): δ 13.08 (s, 1H), 10.26 (s, 1H), 7.61 (d, *J* = 2.0 Hz, 1H), 7.30–7.18 (m, 5H), 7.09–6.98 (m, 4H), 6.84–6.79 (m, 2H), 5.20 (t, *J* = 5.7 Hz, 1H), 3.01 (q, *J* = 6.6 Hz, 2H), 1.37–1.28 (m, 2H), 1.10–1.00 (m, 2H), and 0.74 (t, *J* = 7.3 Hz, 3H).

13C NMR (101 MHz, DMSO-d6): δ 160.37, 157.10, 156.02, 142.92, 139.07, 137.50, 134.35, 129.04 (2C), 128.99 (2C), 123.54, 122.43, 121.22, 119.16 (2C), 115.70 (2C), 112.93, 112.54, 41.90, 29.98, 19.20, and 13.54.
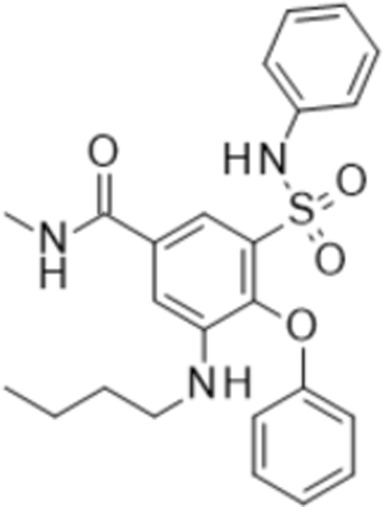


3-(butylamino)-N-methyl-4-phenoxy-5-(phenylsulfamoyl)benzamide (**compound BA-184**).

According to **GP3**, starting from **compound BA-83** and methylamine as a 2M solution in THF, **compound BA-184** was isolated as a white solid (143 mg, 0.299 mmol, 88%).

C24H27N3O4S; MS (ESI+) m/z: 454 [M + H]+; 1H NMR (400 MHz, DMSO-d6) δ 10.14 (s, 1H), 8.56 (q, J = 4.4 Hz, 1H), 7.64 (d, J = 2.0 Hz, 1H), 7.35 (d, J = 2.0 Hz, 1H), 7.31–7.13 (m, 4H), 7.09–6.93 (m, 4H), 6.85–6.76 (m, 2H), 4.86 (t, J = 5.7 Hz, 1H), 3.01 (q, J = 6.5 Hz, 2H), 2.78 (d, J = 4.5 Hz, 3H), 1.37–1.26 (m, 2H), 1.11–0.98 (m, 2H), and 0.73 (t, J = 7.3 Hz, 3H).

13C NMR (101 MHz, DMSO-d6) δ 165.4, 156.2, 142.1, 138.6, 137.7, 133.3, 131.9, 129.0 (2C), 128.9 (2C), 123.3, 122.3, 118.9 (2C), 115.7 (2C), 115.0, 114.0, 41.9, 30.2, 26.3, 19.2, and 13.5.
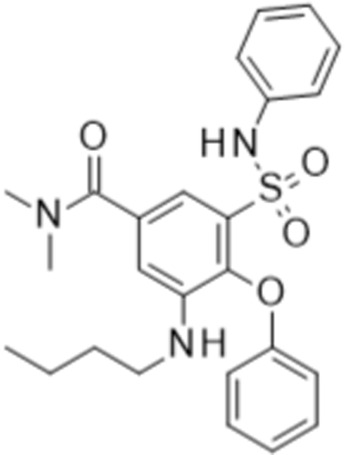


3-(butylamino)-N,N-dimethyl-4-phenoxy-5-(phenylsulfamoyl)benzamide (**compound BA-177**).

According to **GP3**, starting from **compound BA-83** and dimethylamine as a 2 M solution in THF, **compound BA-177** was isolated as a white solid (135 mg, 0.279 mmol, 82%).

C25H29N3O4S; MS (ESI+) m/z: 468 [M + H]+; 1H NMR (400 MHz, DMSO-d6) δ 10.15 (s, 1H), 7.23 (dtd, J = 24.3, 7.3, 1.9 Hz, 4H), 7.09–6.96 (m, 5H), 6.89 (d, J = 1.9 Hz, 1H), 6.85–6.79 (m, 2H), 4.97 (t, J = 5.8 Hz, 1H), 3.06–2.74 (m, 8H), 1.34–1.23 (m, 2H), 1.12–0.98 (m, 2H), and 0.73 (t, J = 7.3 Hz, 3H).

13C NMR (101 MHz, DMSO-d6) 168.9, 156.4, 142.4, 137.7, 137.1, 133.9, 132.9, 129.0 (2C), 128.9 (2C), 123.5, 122.2, 119.2 (2C), 115.6 (2C), 113.9, 113.8, 41.9, 38.8, 34.7, 30.1, 19.2, and 13.6.

### 4.2 NKCC1 function

To assess the effect of the compounds on NKCC1 function, we used native HEK293-T cells (ATCC, CRL3216) plated on 35-mm dishes. NKCC1 function was measured through unidirectional K^+^ influx using radioactive ^83^Rb as a tracer for K^+^ movement. Experiments were performed in a hypertonic saline (370 mOsM) to fully activate the cotransporter. Under these conditions, most of the flux (90%–95%) is mediated by the Na^+^/K^+^ ATPase and NKCC1, with the remainder mediated by K^+^ channels. The flux was performed in the presence of 100 μM ouabain to inhibit the Na^+^/K^+^ pump component and maximize the participation of NKCC1. Two hours prior to the flux, cells were detached from 10-cm dishes and plated on poly-L-lysine (0.1 mg/mL)-coated dishes. We added 2 mL per dish from a homogeneous cell suspension to ensure that all dishes were identical. Typically, one experiment includes eight groups of three dishes (triplicates) for a total of 24 dishes. Groups of three dishes were staggered every 2 min to allow for easy handling and accurate timing. For each group (2 min apart), the culture medium was aspirated and replaced with 1 mL hypertonic saline (130 mM NaCl, 5 mM KCl, 2 mM CaCl_2_, 0.8 mM MgSO_4_, 1 mM glucose, 60 mM sucrose, and 10 mM Na-HEPES; pH 7.4) for a 15-min preincubation period. After preincubation, the saline was aspirated and replaced with 1 mL of an identical solution containing 100 μM ouabain and 0.2 mCi/mL ^83^Rb in the presence or absence of the test compound. After 15-min uptake, the radioactive solution was aspirated, and the dishes were washed thrice with 1 mL ice-cold saline. At the end of the flux, 500 μL of 0.2 N NaOH was added to the dishes for cell lysis, i.e., the release of ^83^Rb and solubilization of proteins. After the lysis period, 250 μL acid acetic glacial was added for neutralization. Aliquots of 200 μL were added to 5-mL scintillation vials to measure counts, and 20 mL aliquots were used for protein assay (BioRad). Aliquots of 5 μL of radioactive solutions used in the flux were also counted to transform cpm into pmoles or nmoles of K^+^. Unidirectional K^+^ influx is, therefore, expressed in pmoles K^+^ per mg protein per min.

### 4.3 Creation of an NKCC2-expressing HEK293 cell line

It is our experience that HEK293 cells do not easily overexpress Na^+^-dependent Cl^−^ cotransporters when NKCC1 is present. Furthermore, NKCC2 function cannot be distinguished from NKCC1 if cells still express it. Thus, we utilized our CRISPR/cas9-generated NKCC1-knockout cell line (Magaña-Ávila et al., 2025) to introduce an NKCC2 cDNA. Human NKCC2 cDNA clone RC216145 was purchased from OriGene™ Technologies. This clone was utilized in a previous study (Savardi et al., 2020). To assess the function, we moved the entire open reading frame to a *Xenopus laevis* oocyte expression vector and demonstrated that that the clone was non-functional. Analysis of the sequence revealed two issues, namely, 1) the ORF contains an unusual exon 5 (96 bp), which makes the transporter “unnatural,” and 2) and, most importantly, it includes a C-terminal epitope tag, Myc-DDK, that disrupts function. After eliminating the epitope tag and restoring the proper sequence of the transporter, function was re-established. The OriGene clone was based on the hNKCC2 cDNA published by Simon et al. (1996). There are three variants of mammalian NKCC2, namely, NKCC2F, NKCC2A, and NKCC2B, due to the alternative splicing of exon 5, which is a cassette exon. The three transcripts are differentially expressed along the nephron, and each sequence confers specific Na^+^ and Cl^−^ binding properties to the transporter. The sequence of exon 5 in the Simon cDNA differs from that of exons 5F, 5A, and 5B in the human genome. To test whether there was another version of the exon, we blasted the human genome with this sequence and failed to identify a match. We also attempted to align the exon with the human gene fragment between exon 4 and 6 and again failed to align the sequence. To avoid any issue, we substituted the unnatural exon with human exon 5A. This clone was then moved to pDNA5 for expression in mammalian cells.

HEK293 cells lacking NKCC1 expression were transfected with the hNKCC2 clone in pCDNA5. Two days after transfection, the cells were placed under hygromycin selection. The cells were then serially diluted in 10-cm culture dishes to isolate individual clones. Clones were picked, grown in 96-well plates, and then tested for the presence of bumetanide-sensitive K^+^ influx. Several positive clones were selected, and clone 16 was used for future experiments. Contrary to NKCC1, for which flux increases under hypertonicity (Gagnon and Delpire, 2010), the hNKCC2-mediated K^+^ influx was not stimulated by hypertonicity, ensuring that the function examined was that of NKCC2 and not NKCC1. These cells were used for testing the effect of a subset of compounds.

### 4.4 KCC2 function

To assess the effect of the compounds on KCC2 function, we used a KCC2-overexpressing cell line created in a previous study (Prael III et al., 2022). To assess KCC2 function, K^+^ influx was measured as described above but using a Na^+^-free saline containing 130 mM Na-NMDG (N-methyl-D-gluconate), 5 mM KNO_3_, 2 mM Ca(NO_3_)_2_, 0.8 mM MgSO_4_, 1 mM glucose, and 10 mM HEPES titrated to pH 7.4 with NMDG.

### 4.5 Molecular docking

For docking, we used the structure of NKCC1 in a complex with bumetanide (PDB: 9c0h). The NKCC1 structure was opened in *PyMOL* (Schrödinger, LLC), and the coordinates of the bumetanide atoms were extracted and averaged. Bumetanide was then deleted, and the structure free of its ligand was saved with a new name. Using the Open Babel chemistry toolbox and SMILES descriptions, *pdb* files were created for each of the ligands. After the addition of partial charges, atom types, and polar hydrogen atoms for both the receptor and ligand molecules (i.e., creation of *pdbqt* files), docking was performed with *Autodock Vina* (The Scripts Research Institute, La Jolla, CA). A python script was written to automatically dock the receptor with each of the ligand, saving both the docking energy log files and *pdbqt* output files in a dedicated folder. The center coordinates were those obtained from bumetanide, the size of the box was set at 10, and the exhaustiveness was set at 3,600. Following docking, the *pdbqt* output files were uploaded to Open Babel, transformed into the *pdb* format, saved, and then opened in pymol, alongside the ligand-free NKCC1 file. A *pdb* file of the complex was then generated and uploaded into PLIP (Protein Ligand Interaction Profiler, https://plip-tool.biotec.tu-dresden.de/) to analyze the atomic interactions.

## Data Availability

The original contributions presented in the study are included in the article/supplementary material; further inquiries can be directed to the corresponding author.

## Glossary

ADMET: Absorption, distribution, metabolism, excretion, and toxicity
ASD: Autism spectrum disorders
CDI: 1,1′-carbonyldiimidazole
CNS: Central nervous system
Cryo-EM: Cryogenic electron microscopy
CV: Column volume
DBU: 2,3,4,6,7,8,9,10-Octahydropyrimidol[1,2-*a*]azepine
DCM: Dichloromethane
DIPEA: Diisopropyl ethylamine
DMA: Dimethylacetamide
DMF: Dimethylformamide
DMF-DMA: *N,N*-dimethylformamide dimethyl acetal
DMSO: Dimethylsulfoxide
Eq.: Equivalent
Equiv.: Equivalent
ESI: Electrospray ionization
EtOAc: Ethyl acetate
EtOH: Ethanol
FDA: Food and Drug Administration (USA)
HATU: 1-[Bis(dimethylamino)methylene]-1H-1,2,3-triazolo[4,5-b]pyridinium 3-oxid hexafluorophosphate
HEK: Human embryonic kidney
HTS: High throughput screening
iPrOH: iso-Propanol
KCC: K–Cl cotransporter
LCMS: Liquid chromatography coupled with mass spectrometry
MeOH: Mmethanol
MS: Mass spectrometry
NKCC: Na–K–2Cl cotransporter
NMR: Nuclear magnetic resonance
Pd/c: Palladium on carbon
PDB: Protein Data Bank
PIDA: Phenyliodonium diacetate
SAR: Structure–activity relationship
**SN**_**AR**_: Nucleophilic aromatic substitution
TBAF: Tetrabutylammonium fluoride
TBDMSCl: tert-Butyl(chloro)dimethylsilane, tert-Butyldimethylchlorosilane
TFAA: Trifluoroacetic anhydride
THF: Terahydrofuran
TLC: Thin-layer chromatography
UV: Ultraviolet
